# Powering the Future: Unveiling the Potential of Na, K, and Mg Solid-State Batteries

**DOI:** 10.3390/nano15110859

**Published:** 2025-06-03

**Authors:** Ruoxu Shang, Yi Ma, Kathrine Anduaga-Quiros, Gustavo Briseno, Yuying Ning, Hung-Ju Chang, Mihrimah Ozkan, Cengiz S. Ozkan

**Affiliations:** 1 Materials Science and Engineering Program, University of California Riverside, Riverside, CA 92521, USA; rshan007@ucr.edu (R.S.); yning012@ucr.edu (Y.N.); mihri@ece.ucr.edu (M.O.); 2 Department of Mechanical Engineering, University of California Riverside, Riverside, CA 92521, USA; yma102@ucr.edu (Y.M.); kquir009@ucr.edu (K.A.-Q.); gbris004@ucr.edu (G.B.); hchan211@ucr.edu (H.-J.C.); 3 Department of Electrical and Computer Engineering, University of California Riverside, Riverside, CA 92521, USA

**Keywords:** solid-state batteries (SSBs), sodium (Na), potassium (K), magnesium (Mg)

## Abstract

In the pursuit of advancing sustainable energy storage solutions, solid-state batteries (SSBs) have emerged as a formidable contender to traditional lithium-ion batteries, distinguished by their superior energy density, augmented safety measures, and improved cyclability. Amid escalating concerns regarding resource scarcity, environmental ramifications, and the safety hazards posed by lithium-ion technologies, the exploration into non-lithium SSBs has emerged as a crucial frontier for technological breakthroughs. This exhaustive review delves into the latest progressions and persisting challenges within the sphere of sodium (Na), potassium (K), and magnesium (Mg) SSBs, spotlighting seminal materials, cutting-edge technologies, and strategic approaches propelling advancements in this vibrant domain. Despite considerable progress, hurdles such as amplifying ionic conductivity, mitigating the intricacies at the electrode–electrolyte interface, and realizing scalable production methodologies continue to loom. Nevertheless, the trajectory for non-lithium SSBs holds considerable promise, poised to redefine the landscape of electric vehicles, portable electronics, and grid stabilization technologies, thereby marking a significant leap toward realizing a sustainable and energy-secure future. This review article aims to provide a detailed overview of the materials and methodologies underpinning the development of these next-generation energy storage devices, underscoring their potential to catalyze a paradigm shift in our approach to energy storage and utilization.

## 1. Introduction

The relentless pursuit of renewable energy sources and the ever-growing hunger for efficient, compact energy storage solutions have ignited a firestorm of innovation in battery technology. Among the many promising contenders, solid-state batteries (SSBs) stand out as beacons of hope, offering tantalizing possibilities for unprecedented energy density, superior safety, and extended lifespans. Beyond the prevalent lithium-ion battery, dedicated research into alternative battery systems is crucial for addressing the multifaceted challenges of a rapidly electrifying world. The primary drivers for this diversified approach include the finite and geographically concentrated nature of lithium and cobalt resources, which pose risks of supply chain instability, price volatility, and geopolitical tensions. Furthermore, the environmental footprint associated with current lithium mining and refining processes, alongside inherent safety concerns like thermal runaway in traditional lithium-ion batteries, underscore the need for safer and more sustainable alternatives. Finally, different applications have varying requirements for energy density, power output, cost, and cycle life, meaning that a “one-size-fits-all” battery solution is insufficient for the diverse demands of grid-scale storage, electric vehicles, and portable electronics.

**Figure 1:**
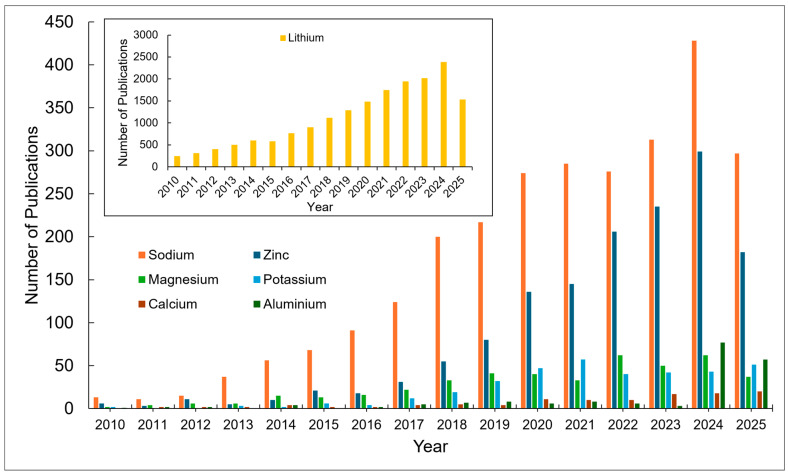
Number of articles related to SSEs published from early 2010 to late 2024. Increasing research trend in the field of solid-state batteries for non-Li SSB systems. The same trend for Li SSB systems from early 2010 to late 2024 is shown in the subset for comparison (Web of Science, searching key words: X + solid + state + battery, X = Li, Na, Zn, Mg, Ca, Al).

This article delves deep into the exciting world of non-lithium solid-state batteries, offering a comprehensive overview of the latest advancements and challenges. Figure 1 summarizes the annual publication of articles on lithium [1,2,3,4,5,6] and various non-lithium solid-state batteries [7,8,9] from early 2010 to early 2025. It is evident that while solid-state lithium batteries dominate, there has been growing interest in non-lithium solid-state batteries each year, especially in sodium, potassium, and magnesium batteries. Compared with solid-state lithium batteries, these batteries exhibit advantages and unique characteristics. Among the promising alternatives, Na-ion batteries leverage Earth’s abundant and low-cost sodium reserves, offering enhanced safety due to a reduced risk of thermal runaway and the ability to withstand over-discharge, along with good low-temperature performance [10]. Their main disadvantage is a lower energy density compared with lithium-ion batteries, making them more suitable for stationary grid energy storage, backup power systems, and low-speed electric vehicles where weight and volume are less critical. K-ion batteries also benefit from the high abundance and low cost of potassium. They exhibit potentially faster charging capabilities due to the larger ionic radius of potassium, which facilitates faster diffusion in certain electrode materials, and can operate across a wide temperature range [11]. However, they typically suffer from a lower energy density and less stable electrode materials compared with lithium, leading to a shorter cycle life in current prototypes. Consequently, K-ion batteries are being explored for grid-scale storage, specific industrial applications, and potentially even some consumer electronics where rapid charging is prioritized over ultimate energy density. Finally, Mg-ion batteries capitalize on the exceptional abundance and low cost of magnesium, with the theoretical advantage of higher volumetric energy density due to the divalent nature of Mg^2+^ ions [12]. They also offer inherent safety advantages by being less prone to dendrite formation than lithium. The major hurdles for Mg-ion batteries include slow ion diffusion kinetics due to strong electrostatic interactions and the limited availability of suitable electrolytes and cathode materials, placing them in an earlier stage of development, primarily targeted for future electric vehicles and stationary storage, if these fundamental challenges are overcome. We unveil the key materials, groundbreaking technologies, and innovative design strategies that are powering this revolution. For example, Yang et al. developed an ultra-stable composite sodium anode, incorporating well-dispersed ion-conductive Na_x_TiO_2_ to stabilize the anode interface of a Na_3_Zr_2_Si_2_PO_12_ electrolyte, resulting in an ultra-low interfacial impedance, high critical current density, and stable plating/stripping performance lasting over 8000 h at 0.1 mA cm^−2^ [13]. For another example, Du et al. prepared a K-ion composite electrolyte membrane that demonstrated exceptional electrochemical stability, mechanical flexibility, and high ionic conductivity (9.31 × 10^−5^ S cm^−1^ at 25 °C), showing superior electrochemical performance even at low temperatures (99.7 mAh g^−1^ at −20 °C) [14]. However, the path to widespread adoption is fraught with hurdles. We delve into the major obstacles that these batteries face including limitations in ionic conductivity, complexities at the electrode–electrolyte interface, and the need for scalable manufacturing processes.

Despite these challenges, the prospects for non-lithium solid-state batteries remain highly promising. We explore their potential applications, ranging from powering the next generation of electric vehicles to revolutionizing portable electronics and stabilizing our increasingly renewable energy grids. For instance, Lin et al. recently discovered a new sodium superionic glass, 0.5Na_2_O_2_-TaCl_5_, featuring a dual-anion sublattice of oxychlorides. The assembled Na-ion solid state battery exhibits ultrahigh ionic conductivity (4.62 mS cm^−1^ at 25 °C) and ensures a favorable electrolyte–electrode interface, leading to superior cycling stability for over 500 cycles at room temperature [15]. Ma et al. developed a solid-state Mg hybrid ion full battery capable of withstanding temperatures ranging from −20 °C to 120 °C and enduring 180 sewing test cycles. The batteries exhibited excellent electrochemical performance and superior cyclic stability of 95.9% after 5000 cycles at 2 A⋅g^−1^, marking a significant step toward practical applications in grid-scale energy storage and flexible/wearable devices [16]. We identify the crucial technological breakthroughs needed to unlock their full commercial potential, paving the way for a more sustainable and energy-secure future. We also strongly urge all sectors of society to take action, continue to pay attention to research and development in the field of non-solid-state batteries, and contribute their efforts to achieve environmental goals for green emissions reduction.

## 2. Na-Ion Solid-State Battery Systems: Progress and Challenges

In pursuit of safer and more sustainable battery technologies, research on sodium-ion solid-state batteries (SSSBs) has gained significant momentum. SSSBs hold the potential to address the safety concerns associated with traditional lithium-ion batteries (LIBs) while utilizing a more abundant and cost-effective element, sodium. However, several key challenges remain including the development of high-performance solid-state electrolytes (SSEs), optimizing electrode-electrolyte interfaces, and mitigating side reactions [17]. Various SSEs have been proposed as potential solutions. In 2014, a significant milestone was reached with the development of the first all-solid-state sodium-ion battery featuring an inorganic sodium layered oxide cathode. Despite this innovation, the system encountered challenges, notably a decline in capacity attributed to escalating cell resistance through successive cycles [18]. To address this concern, researchers have broadened their investigations to include both organic and hybrid solid electrolytes, aiming to enhance performance and mitigate resistance issues. In 2018, researchers successfully synthesized two types of electrolytes: an organic gel electrolyte and a hybrid solid polymer nanocomposite electrolyte. Guo and colleagues focused on a gel polymer electrolyte formulated from poly(vinylidene fluoride-co-hexafluoropropylene) (P(VDF-HFP)). They evaluated its performance in an electrochemical cell, pairing Na_3_V_2_(PO_4_)_3_F_3_ as the cathode with an anode made from hard carbon derived from cotton cloth. The GPE was crafted by dissolving the P(VDF-HFP) polymer in a mixture of N,N-dimethylformamide (DMF) and distilled water, followed by a process of casting and drying. This preparation resulted in a gel electrolyte that demonstrated a stable capacity of 120 mAh g^−1^ over 1500 cycles in a Na_3_V_2_(PO_4_)_3_F_3_/Na half-cell configuration [19]. Ni’mah et al. developed an innovative solid polymer electrolyte (SPE) nanocomposite, where they utilized polyethylene glycol (PEO), sodium perchlorate (NaClO_4_), and titanium dioxide (TiO_2_) [20]. They developed two types of electrolytes, PEO/NaClO_4_ and a nanocomposite of TiO_2_/PEO/NaClO_4_, employing solution casting techniques for their preparation. The nanocomposite electrolyte TiO_2_/PEO/NaClO_4_ exhibited enhanced ionic conductivity, reaching up to 2.62 × 10^−4^ S cm^−1^ at 60 °C. The electrochemical performance of this nanocomposite polymer electrolyte was thoroughly evaluated, showcasing commendable stability with a discharge capacity of 45 mAh g^−1^ over 25 cycles [20].

### 2.1. Cathode Considerations in the Context of Na-Ion Batteries

In response to the urgent challenges posed by climate change and the dwindling availability of fossil fuels, the development of large-scale energy storage systems has become imperative. Among the various energy storage technologies, electrochemical secondary batteries stand out due to their versatility, high energy conversion efficiency, and straightforward maintenance, making them an attractive option for large-scale electricity storage [21]. Since their commercialization by Sony in the early 1990s, LIBs have dominated the portable electronic market. However, the escalating demand for lithium, spurred by these new, large-scale applications, is expected to significantly drive-up lithium prices and strain the global lithium reserves. Given that lithium is not abundantly available in nature, this trend poses a sustainability challenge. For instance, global lithium consumption in 2008 was estimated at approximately 21,280 tons, and increased to nearly 180,000 tons in 2023 [22]. With an average compound annual growth rate of 13.9%, the current exploitable lithium resources are projected to last only around 65 years, complicating and potentially making the broad implementation of such technologies prohibitively expensive. In contrast, sodium is the fourth most abundant element on Earth; continental resources of salt are vast, and the salt content in the oceans is nearly unlimited. Resources of rock salt and salt from brine are primarily in Kansas, Louisiana, Michigan, New York, Ohio, and Texas in the United States. Almost every country in the world has salt deposits and solar evaporation operations of various sizes. Sodium-ion batteries [23,24], operating on the same principle as LIBs, promise an easier and less costly transition, given the potential for technology transfer from LIB manufacturing processes to sodium-ion battery production. The cathode material plays a crucial role in determining the electrochemical performance of sodium-ion batteries. Significant research efforts have been directed toward exploring cathode materials including layered transition metal oxides (LTMOs), polyanionic compounds, and Prussian blue and its analogues (PBA) [25]. These materials are evaluated based on their conductivity, kinetic properties, and cycling performance. Table 1 presents a comparative analysis of the electrical performance of these cathode materials, highlighting the ongoing efforts to optimize sodium-ion battery technology for sustainable, large-scale energy storage solutions. Table 1 showcases the electrical performance of these materials.

#### 2.1.1. Layered Structures of P2 and O3-Type NaTMO_2_ Cathode Materials

**Figure 2:**
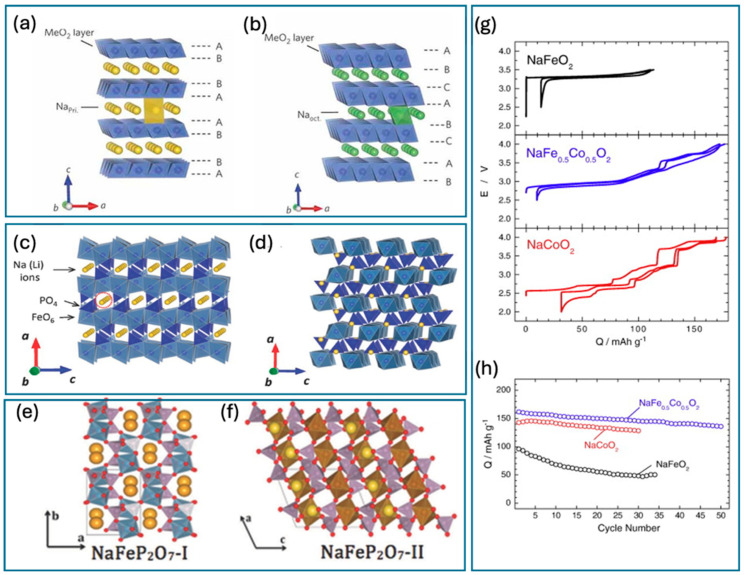
(**a**) Layered structures of the P2- and (**b**) O3-type NaTMO_2_. Reprinted with permission. Copyright 2012, Nature Publishing Group [42]. A, B, and C represent three distinct positions that oxygen layers can occupy in closed packed stacking. MeO_2_ layers are layers of transition metal (Fe/Mn) oxides, whereas Na_pri._ and Na_oct._ refer to sodium ions occupying prismatic and octahedral sites, respectively, between the MeO_2_ layers. (**c**) Crystal structures of phosphate-based compounds with Fe: triphylite-type Na(Li)FePO_4_ and (**d**) maricite-type NaFePO_4_. Reproduced with permission. Copyright 2014, American Chemical Society [43]. (**e**) The rich crystal chemistry observed in sodium metal pyrophosphates. Monoclinic structured polymorphs of I-NaFeP_2_O_7_ and (**f**) II-NaFeP_2_O_7_. Reproduced with permission [43]. (**g**) Charge/discharge curves of NaFeO_2_, NaFe_0.5_Co_0.5_O_2_, and NaCoO_2_ in Na cells at a rate of 12 mA g^−1^. (**h**) The changes in the discharge capacity for 50 cycles. Reprinted with permission. Copyright 2013, Elsevier Publishing Group [44].

LTMOs, particularly those based on manganese, have garnered attention for their substantial reversible capacities due to their relatively modest molecular weights. Utilizing LTMOs based on abundant and environmentally benign elements can contribute to the development of more sustainable battery technologies, reducing reliance on scarce or toxic materials. This class of materials was first identified as potential cathode materials in 1971 by Parent et al., and are characterized by the general formula Na_x_MnO_2_ [38]. These compounds exhibit a layered architecture conducive to various structural transformations across distinct voltage ranges, indicative of their unique electrochemical properties. A mixture of tunnel and layered structures forms between 0.22 ≤ x ≤ 0.44 V, 0.66 < x ≤ 1 V, and 0.44 < x ≤ 0.66 V. The classification of LTMOs into types such as P2, P3, and O3 is predicated on the positioning of alkali ions, with this discourse concentrating on P2 and O3 phases—where ‘O’ signifies the octahedral positioning of Na ions and ‘P’ represents their trigonal positioning. These categorizations are further delineated by the structural packing sequences of TMO_6_ octahedra, highlighting the nuanced electrochemical frameworks these materials offer. The numbers 2 or 3 refer to the count of the shared-edge TMO_6_ octahedra of oxygen arranged in either ABBA or ABCABC packing sequences, respectively (Figure 2). While LTMOs, particularly manganese-based varieties, exhibit promising characteristics for sodium-ion batteries, further research efforts are needed to unlock their full potential and address the existing limitations. Here are some key future directions for this field [39,40,41], outlined as follows.

(i) Optimizing structural stability and reversible capacity

Exploring novel dopants and substitutions:

Introducing dopants like cobalt and nickel, or partially substituting metals in P2 and O3 LTMOs for Na-ion batteries, could enhance structural stability and reduce voltage fade [17,18,19]. These modifications aim to improve the performance and lifespan of Na-ion batteries. Substituting specific dopants can tailor interionic bond lengths for a more robust structure against cycling stresses. Adjustments to the electronic structure can stabilize transition metal valence states, preventing phase changes that cause voltage fade. Computational methods like density functional theory (DFT) can model these changes, guiding the design of effective modifications by predicting stability, conductivity, and ion diffusion [3,4,5].

Tailoring crystallographic parameters:

Optimizing crystallographic lattice parameters by adjusting the Na content can minimize structural transformations and capacity fade in sodium-ion batteries. This focuses on creating materials with crystal structures that reduce undesirable Jahn–Teller distortions during Na insertion/extraction [11,12,13]. Modifying these parameters to better accommodate Na ions leads to more stable structures during cycling, enhancing cyclability and longevity. Reducing Jahn–Teller distortions facilitates smoother Na ion diffusion. DFT can predict how structural modifications affect battery performance, guiding the synthesis of improved materials [12,18].

Understanding and mitigating phase transitions:

Phase transitions can degrade the battery capacity and stability. Surface modification and nanocomposite formation are promising mitigation strategies. Surface modification improves the interaction with Na ions and electrolytes, enhancing performance. Engineering the surface, possibly with thin films or dopants, could minimize structural changes and stabilize electronic properties, improving the electrode–electrolyte interface and reducing kinetic barriers for Na ion diffusion [12,19].

(ii) Enhancing rate capability and electronic conductivity

Engineering nanostructures:

Fabricating LTMOs with controlled morphologies, like nanoparticles, nanorods, or nanosheets, strategically enhances the Na-ion battery performance. These forms optimize the electrode–electrolyte interface and ion transport. Nanoparticles increase the contact area for efficient electron and ion exchange. Nanorods and nanosheets offer large surface area and structural coherence, providing more active sites and enhancing charge storage [17,29]. Nano-structuring creates direct Na^+^ ion diffusion pathways, improving the rate capability. Nanoparticles suit high power density, while nanorods/sheets may be better for high energy density and stability.

Composite electrode design:

Incorporating LTMOs into conductive matrices like carbon frameworks or conducting polymers can create composites with improved electronic conductivity and faster charge/discharge. Carbon materials (graphene, carbon nanotubes, carbon fibers) provide a conductive network enhancing electron transfer [9,10,11]. Conducting polymers can also serve as matrices, contributing to conductivity. The choice depends on the desired properties. Embedding LTMOs in a conductive matrix significantly reduces the internal resistance, enabling faster rates. The matrix’s porous structure can also facilitate rapid Na^+^ ion transport.

Interface engineering:

Optimizing the electrode–electrolyte interface via surface functionalization or coatings can facilitate Na^+^ ion transport and reduce interfacial resistances, improving the rate performance. This interface is critical for electrochemical reactions, affecting efficiency and lifespan. Functional groups can provide pathways for smoother Na+ ion transport, reducing energy barriers. Improved wettability from functionalization enhances electrolyte contact, lowering resistance [21,23,24,25,29]. Protective coatings can prevent degradation and side reactions. Ionically conductive coatings can enhance interface conductivity, ensuring efficient Na+ ion movement. Stable interphase layers maintain interface integrity under demanding conditions.

(iii) Exploring novel LTMO compositions and structures

Investigating beyond P2 and O3 phases:

Exploring alternative LTMO structures like layered Ruddlesden–Popper phases or double-layered perovskites may offer better stability and electrochemical behavior. Ruddlesden–Popper phases, with alternating oxide and rock salt layers, facilitate Na ion intercalation/deintercalation [39,40,41]. Their inherent stability mitigates volume changes and stresses, improving cyclic stability. Defined ion migration pathways enhance rate capability. Compositional tailoring allows for the optimization of electrochemical properties. Double-layered perovskites, with a complex oxide framework, easily accommodate Na ions. Their open framework allows for efficient ion transport, contributing to high ionic conductivity and charge/discharge rates. Both structures offer compositional flexibility for fine-tuning properties and can withstand cycling stresses, reducing capacity fade [11,15,23].

Designing high-voltage materials:

Identifying new LTMO compositions for higher operating voltages in Na-ion batteries is crucial for higher energy densities. Manipulating transition metal oxidation states or incorporating redox-active elements can significantly enhance the electrochemical performance [11,41]. Adjusting oxidation states can optimize the electrochemical window for higher voltages. Selecting metals with suitable redox potentials allows for reversible processes without structural compromise. Multi-electron redox reactions enable more charge storage at elevated voltages. Redox-active elements (Mn, Fe, Co, V) can also contribute to higher voltages and capacities [1,2,3,4,5]. However, the chemical and thermal stability of LTMOs must be considered at higher voltages.

Combining computational and experimental approaches:

Utilizing computational tools, particularly DFT, to predict novel LTMO structures for Na-ion batteries is a leading-edge approach. DFT accurately predicts electronic and structural properties like band structure and lattice parameters, crucial for determining a material’s suitability as a cathode. Computational modeling can identify optimal Na-ion diffusion pathways, essential for high-rate performance. DFT calculations can be used in high-throughput screening to rapidly evaluate potential cathode materials. Simulating compositional variations can predict their impact on performance, leading to the discovery of enhanced LTMOs [23,24,25,29,33,36]. Computational predictions serve as a benchmark for experimental validation, refining DFT models for future studies.

By pursuing these research directions, the development of LTMOs as high-performance cathode materials for Na-ion batteries can be advanced, contributing to efficient and sustainable energy storage solutions.

##### P2-Type Metal Oxide Cathode Materials

P2-type LTMOs (Na_x_TMO_2_; TM = Mn, Co, Ni, Ti, Fe, V, Cr, or combinations) present themselves as compelling cathode materials for sodium-ion batteries due to their favorable Na^+^ ion conductivity and structural stability. This is primarily attributed to the P2 structure offering rapid Na+ diffusion compared with the O3 framework, characterized by low-energy conduction pathways [45]. Notably, in P2-type materials, Na+ ions reside within triangular prism sites and navigate directly through shared rectangular faces, exhibiting low activation energy barriers for inter-site hopping. A prime example is the layered oxide Na_0.78_Ni_0.23_Mn_0.69_O_2_ [46] synthesized by Ma et al., boasting a high energy density attributed to its initial P2 phase [21]. Despite their inherent advantages, including high capacity, certain limitations impede their industrial implementation. Firstly, P2-type metal oxides exhibit a modest initial discharge capacity of around 80 mAh g^−1^ below 4.0 V [24]. Additionally, they are susceptible to phase transitions during charge/discharge cycles, transforming into O2 or OP4/‘Z’ phases, thereby compromising material performance [25]. For instance, in Na_x_[Ni_1/3_Mn_2/3_]O_2_, the P2 phase persists for 1/3 ≤ x ≤ 2/3 but transitions to the O2 phase below x = 1/3 [47]. The fluctuating TMO_2_ panels and specific voltage ranges can trigger structural collapse and capacity fade, highlighting the inherent trade-off between capacity and stability. However, incorporating additional elements can enhance structural stability and potentially increase capacity without inducing phase transitions, offering an avenue for improvement [38]. Another key challenge lies in the low Coulombic efficiency exhibited by P2-type materials during the first cycle in half-cell configurations [42]. To address this issue, Ilarduya et al. proposed incorporating NaN_3_ to compensate for the sodium deficiency inherent to P2-type cathodes [9]. While their experiments with additions of 10% and 20% NaN_3_ yielded positive results, practical implementation remains hindered by the difficulty of integrating NaN_3_ into industrial synthesis processes involving noble metals [48]. To bridge the gap between research and real-world applications, future research efforts should focus on the following. The first is TM layer composition optimization, which involves identifying specific TM combinations that hinder phase transitions, effectively enhances structural stability. The second aspect is the stabilization of anionic redox reactions, which would involve exploring strategies to stabilize the anionic redox process, potentially improving the overall electrochemical performance. By addressing these critical challenges, P2-type layered transition-metal oxides hold immense potential to revolutionize the landscape of cathode materials, paving the way for efficient and sustainable energy storage solutions.

##### O3-Type Metal Oxide Cathode Materials

Compared with their P2-type counterparts, O3-type layered transition-metal oxides offer a high energy density and wider voltage windows (2.2–4.5 V) but suffer from limitations in working potential [49]. Notably, these materials can accommodate a 1:1 Na:M stoichiometry. While α-NaFeO_2_ pioneered their use in LIBs and later in sodium-ion batteries [50], its structure degrades readily due to Fe migration, leading to rapid capacity decline [44]. The key challenge arises from the transformation of triangular prism sites in O3-type structures to octahedral sites. Inter-site hopping for Na^+^ ions requires a metastable tetrahedral intermediate, incurring a high activation energy barrier attributed to the octahedral-tetrahedral face. To address this issue, researchers have explored partial Fe substitution with alternative transition metals like cobalt, manganese, and nickel within the NaFeO_2_ structure. The voltage profiles and cycling performance are shown in Figure 2g,h [44]. Jang-Yeon et al. demonstrated a well-balanced O3-type material, Na_0.32_[Fe_0.13_Mn_0.40_]O_2_, combining favorable aspects of Na[Ni_0.25_Fe_0.25_Mn_0.25_]O_2_ and Na[Ni_0.4_Co_0.3_Mn_0.3_]O_2_, and achieved a high specific capacity, excellent cycling stability, and superior rate capability [51].

Recently, Ji-Li Yue et al. introduced a novel quaternary O3-type material, Na(NiCoFeTi)_1/4_O_2_, which exhibited an exceptional rate capability and long cycle life at room temperature [14]. Notably, their simple solid-state reaction yielded a material with a remarkable first-cycle reversible capacity of 116 mAh g^−1^ and maintained 93.03% capacity after 100 cycles at a 1 C rate. At a high rate of 20 C, it delivered a reversible capacity of 90.6 mAh g^−1^ and retained 75.0% of its initial capacity after 400 cycles at 5 C. This impressive performance was attributed to Ti substitution, smoothing charge/discharge plateaus, and potentially mitigating Fe migration [52]. Vassilaras et al. compared the electrochemical properties and structural transitions of NaNi0_.5_Co_0.5_O_2_ and NaNi_0.5_Fe_0.5_O_2_ [53]. While the former exhibited multiple phase transitions, the latter only underwent the typical O3–P3 transition. Additionally, higher temperature cycling revealed more Na extraction/reinsertion in NaNi_0.5_Co_0.5_O_2_, suggesting rate limitations. Optimizing and minimizing irreversible structural changes during charging significantly reduced the capacity fade in NaNi_0.5_Fe_0.5_O_2_. Research further suggests instability in Mn-containing Na-TM-oxides. To address this challenge, Bachu Sravan Kumar et al. designed a composition combining TM and non-TM ions, significantly improving the air and water stability. This meticulous selection of ions effectively suppressed phase transformations during electrochemical cycling [54]. Furthermore, Julia Lamb et al. established a molten-salt synthesis technique to prepare layered O3-type Na(Ni_0.3_Fe_0.4_Mn_0.3_)O_2_ single-crystals. This method eliminated the need for co-precipitated precursors, resulting in enhanced morphology, improved cycle life, and increased air stability. While offering high energy density and wider voltage windows, O3-type cathode materials face challenges regarding the working potential, Fe migration, and structural stability. Research efforts have focused on transition metal substitution, novel material compositions, and advanced synthesis techniques hold immense promise for overcoming these limitations and unlocking the full potential of O3-type materials for high-performance Na-ion batteries.

##### Polyanion-Type Cathode Materials

Compared with layered transition metal oxides, polyanion-type materials offer several advantages for sodium-ion batteries. The presence of polyanion groups leads to a higher operating potential due to their inductive effect [55]. Additionally, their robust 3D framework significantly reduces structural variations during sodium ion de/intercalation, improving cycle stability [18]. Furthermore, the strong X-O (X = S, P, Si, etc.) covalent bonds effectively inhibit oxygen evolution, enhancing safety. These combined advantages translate to superior cycle stability and high safety for polyanion-type materials, making them attractive candidates for cathode applications. However, limitations hinder their widespread adoption. One key challenge is low electronic conductivity, which impedes efficient charge/discharge processes [55]. Additionally, the limited capacity compared with conventional materials restricts their energy density. Overcoming these limitations is crucial for the broader application of polyanion-type cathodes. Polyanion-type electrode materials, defined as compounds containing tetrahedral anion units (XO_4_)_n_^−^ or their derivatives (X_m_O_3m+1_)_n_^−^ (X = S, P, Si, As, Mo, or W), are characterized by strong covalent-bonded MO_x_ polyhedra (M representing a transition metal) [56]. Based on the type of anion group, they can be categorized into five main groups: silicates, phosphates, pyrophosphates, sulfates, and mixed-polyanion compounds [57].

##### Phosphate-Based Cathode Materials

Within the polyanion-type electrode materials category, NaFePO_4_ (M = Fe, Mn) has garnered substantial interest for use in sodium-ion batteries due to its pioneering research and comprehensive characterization [43]. This discussion specifically examines the iron-based variant (M = Fe). Notably, NaFePO_4_ is known to exhibit two distinct structural phases: triphylite and maricite. Triphylite-NaFePO_4_ features a one-dimensional (1D) channel that facilitates the transport of Na^+^, as illustrated in Figure 2c, whereas maricite-NaFePO_4_ lacks such structural channels, as shown in Figure 2d. These structural variations significantly influence the synthesis strategies employed. Traditional high-temperature solid-state reactions generally promote the formation of maricite-NaFePO_4_, which is less desirable for applications requiring efficient ion transport. To overcome this limitation, researchers have developed methods such as chemical or electrochemical displacement, often using organic solutions, to transform triphylite-LiFePO_4_ (its lithium-ion counterpart) into the sodium-based variant. NaFePO_4_ is recognized for its impressive cycling stability, rivaling that of LiFePO_4_, a benchmark material in battery technology. However, its rate performance is significantly constrained by a low Na^+^ diffusion coefficient [58]. This inherent property of NaFePO_4_ slows down the movement of Na^+^ ions within the crystal lattice, which is a critical factor in determining the speed at which the battery can charge and discharge. As a result, despite its ability to retain about 90% of its capacity over 100 charge–discharge cycles at a modest rate of 0.1 C, NaFePO_4_ struggles to meet the demands of high-power applications. This limitation is a notable drawback in contexts where rapid charging or high output intensity is required, highlighting the need for further materials engineering to enhance the ionic conductivity without compromising the material’s inherent stability.

##### Pyrophosphates for Cathode Materials

Na-based pyrophosphates such as NaMP_2_O_7_ (M = Ti, V, Fe), Na_2_MP_2_O_7_ (M = Fe, Mn, Co), and Na_4_M_3_(PO_4_)_2_P_2_O_7_ (M = Fe, Co, Mn) are garnering significant research interest for their application in sodium-ion batteries given their structurally diverse and chemically robust frameworks. These compounds are particularly valued for their stable chemical structures, which can efficiently host and shuttle sodium ions during the battery’s charge and discharge cycles. The diverse valence states of the transition metals (Ti, V, Fe, Mn, Co) in these pyrophosphates allow for various redox reactions, contributing to their promising electrochemical properties. These properties include improved ionic conductivity and high theoretical capacities, which are critical for improving the energy density and efficiency of sodium-ion batteries. Additionally, the ability to tailor the electrochemical properties of these materials by substituting different transition metals in the crystal lattice offers the potential to optimize and enhance battery performance under various operating conditions. As shown in Figure 2 and Table 2, NaFeP_2_O_7_ exhibits two distinct phases: thermally unstable I-NaFeP_2_O_7_ (Figure 2e) and the more stable crystalline II-NaFeP_2_O_7_ (Figure 2f), which forms above 750 °C [43]. Similar pyrophosphates, NaTiP_2_O_7_ and NaVP_2_O_7_, have also been reported. Notably, Kee et al. demonstrated the feasibility of NaVP_2_O_7_ as a high-voltage cathode material (3.4 V) for sodium-ion batteries [59]. On the other hand, the Na_2_MP_2_O_7_ family of materials can be categorized into three structural types: triclinic, tetragonal, and monoclinic. The specific structures and properties vary depending on the transition metal (M) [33]. Notably, Na_2_MnP_2_O_7_ showed a superior performance compared with its lithium counterpart, Li_2_MnP_2_O_7_ [42] and exhibited a reversible capacity of 90 mAh g^−1^ at 3.8 V, retaining 96% capacity after 30 cycles and 70% at a higher rate (1 C) [60]. However, its intrinsic low electronic conductivity limits its rate capability. Substituting Mn with Fe partially addressed this issue, enhancing battery performance [61].

#### 2.1.2. Transition Metal Sulfates Na_x_M_y_(SO_4_)z (M = Fe, Mn, Co, Ni)

Transition metal sulfates, represented by the formula Na_x_M_y_(SO_4_)_z_ (where M = Fe, Mn, Co, Ni), have gained attention as promising cathode materials for sodium-ion batteries due to their distinct advantages. These materials stand out because of their strong electronegativity and high redox potentials compared with other polyanionic compounds, which can contribute to potentially higher energy densities [60]. The electronegativity of sulfates allows for the stabilization of the transition metal oxidation states, enhancing the battery’s overall energy output. Additionally, the sulfate groups can induce structural benefits that improve the overall electronic conductivity and ionic transport within the battery, further boosting the energy capacity and efficiency. Na_2_Fe_2_(SO_4_)_3_ stands out among the sodium-ion battery cathode materials for its superior electrochemical properties, attributable to its alluaudite-type structure. This structure features edge-sharing FeO_6_ octahedra linked by SO_4_ units, creating a robust three-dimensional framework with spacious tunnels along the c-axis. These tunnels facilitate the easy insertion and extraction of Na+ ions, crucial for efficient battery operation. Moreover, the Fe^3+^/Fe^2+^ redox couple in Na_2_Fe_2_(SO_4_)_3_ provides an impressive operating potential of 3.8 V, the highest reported for Fe-based cathode materials to date [63]. This high voltage is significant as it enhances the energy density of the battery, making Na_2_Fe_2_(SO_4_)_3_ an excellent candidate for advanced energy storage applications where high performance and reliability are essential.

#### 2.1.3. Transition Metal Silicates Na_2_MSiO_4_ (M = Fe, Mn, Co)

Orthosilicate transition metal silicates, specifically those with the formula Na_2_MSiO_4_ (where M = Fe, Mn, Co), are garnering considerable attention as cathode materials for sodium-ion batteries. These materials are not only abundant and environmentally benign, making them sustainable choices for battery production, but they also exhibit compelling electrochemical properties that are advantageous for many applications. Their structure allows for stable cycling and good ionic conductivity, which are critical for efficient energy storage systems. Furthermore, the presence of silicon in these compounds contributes to a more robust structural integrity and potentially enhances electrochemical stability and capacity retention during prolonged battery operation. Li et al. demonstrated the successful synthesis of Na_2_FeSiO_4_ using both solid-state and sol–gel methods, achieving a reversible capacity of 106 mAh g^−1^ at 30 °C within a voltage window of 1.5–4.0 V [44]. Notably, a clear plateau at 1.8 V was attributed to the Fe^2+^/Fe^3+^ redox reaction. Remarkably, the volume shrinkage of Na_2_FeSiO_4_ during charging only reached 0.5, 0.6, and 0.9% at 2.5, 3.5, and 4.0 V, respectively, highlighting its superior dimensional stability compared with other polyanionic materials [64]. Despite these promising findings, significant challenges remain. Preventing the formation of undesired sodium silicate phases (e.g., Na_2_SiO) and mitigating the oxidation of Fe^2+^ to Fe^3+^ are crucial concerns requiring further investigation [44]. Additionally, strategies to enhance its overall electrochemical performance are necessary to fully unlock its potential. Other polyanion-type compounds have also gained attention such as carbonophosphates [65], amorphous polyanion compounds, and molybdenates [66].

#### 2.1.4. Prussian Blue (PB) and Its Analogues (PBAs)

PBAs have garnered significant interest as cathode materials for Na-ion batteries due to their unique properties. This discourse explores the key characteristics of PBAs including their low cost, simple synthesis, large interstitial spaces, and diverse redox-active sites. Additionally, it examines the limitations of PBAs, particularly the presence of Fe(CN)_6_ vacancies and their impact on electrochemical performance. During the materials’ synthesis process, there is always an Fe(CN)_6_ vacancy produced, which will then be filled with coordinated water. With these two, the chemical formula form of PB and PBAs change from the ideal NaxMFe(CN)_6_ with a perfect lattice to practical NaxM[Fe(CN)_6_]y·□_1−y_·nH_2_O (where □ stands for the Fe(CN)_6_ vacancies) [67]. The section concludes by highlighting the current research efforts toward mitigating these limitations and enhancing the overall cyclability of PBAs.

(i) Advantages of PBAs

PBAs, composed primarily of economically viable elements like iron (Fe) and manganese (Mn), are gaining attention as cost-effective cathode materials for sodium-ion batteries [68]. These materials stand out not only for their affordability, but also for their straightforward synthesis. Employing methods such as hydrothermal synthesis or precipitation, PBAs can be produced on a large scale, making them suitable for commercial applications. The unique crystal structure of PBAs, characterized by large interstitial spaces that exceed the size of sodium ions, allows for the easy movement of these ions. This facilitates efficient insertion and extraction processes, which are critical for the high performance of the batteries [69]. Furthermore, the versatility in the electrochemical behavior of PBAs stems from the different combinations of transition metals that can occupy the M1 and M2 sites within their framework. This structural variability allows PBAs to support either dual-electron transfer or single-electron transfer mechanisms. Such flexibility is instrumental in customizing the theoretical capacities of PBAs to suit specific energy requirements, enhancing their functionality across various energy storage technologies [70]. This adaptability, combined with cost-effectiveness and ease of synthesis, positions PBAs as a promising option in the evolving landscape of battery materials.

(ii) Limitations of PBAs

During the synthesis of PBAs, the formation of vacancies in the Fe(CN)_6_ framework is a common, albeit undesirable, phenomenon. These vacancies are typically filled with water molecules, leading to structural deviations from the ideal crystalline composition. This alteration impacts the electrochemical performance of PBAs by influencing their ion exchange capacity and electronic conductivity. The introduction of water molecules into these vacancies can result in a less stable crystal lattice, which is prone to structural degradation during electrochemical cycling. This degradation manifests as irreversible structural transitions, such as lattice distortion or partial dissolution, which severely limit the cycle life of the battery. Studies have shown that these structural instabilities significantly contribute to the poor cyclability of PBAs, resulting in decreased efficiency and shortened battery life over repeated charge–discharge cycles [71,72,73]. Understanding and mitigating these defects through advanced synthesis techniques or materials engineering is crucial for enhancing the durability and performance of PBAs in practical applications.

#### 2.1.5. Other Types of Cathode Materials

Beyond the commonly discussed families of transition metal oxides, phosphates, and pyrophosphates, a diverse range of alternative cathode materials holds promise for further advancing sodium-ion batteries. This section delves into these less-explored avenues, highlighting their advantages, limitations, and future research directions.

##### NASICON-Based Compounds

NASICON-type materials (sodium superionic conductors) offer intriguing possibilities due to their open 3D framework structures, facilitating Na^+^ ion insertion/extraction. NASICONs with the AMM*P_3_O_12_ (A = Li^+^, Na^+^, K^+^, etc.) general formula show a typical 3D framework. MM*O_6_ octahedra and PO_4_ tetrahedra share the oxygen corners, and the interstitial space in the 3D framework forms interconnected channels for mobile cations. All such compounds with the same 3D topology are called NASICONs, with examples including the Na_1+x_Zr_2_P_3−x_Si_x_O_12_ system of compounds. While their working potential (~2.7–3.0 V) is relatively low, they possess theoretical capacities exceeding 200 mAh g^−1^, leading to potentially high specific energies (>600 Wh kg^−1^) [74]. NASICONs exhibit a high stability to air and moisture while the poor interface contacts resulting in voltage fading are the main challenges [68].

##### Organic Compounds

Organic molecules offer unique advantages due to their structural flexibility and chemical tunability. This allows for tailoring a wide range of operating potentials and capacities [69]. However, their practical application is often hindered by limitations in cyclability and rate capability. Further research is needed to improve their stability and durability.

##### Hexacyanometalates

Materials such as Prussian blue analogues are distinguished by their unique redox behavior and structural diversity, offering high versatility in tunable operating potentials and capacities [75]. Despite their potential, challenges persist in enhancing their cyclability and addressing the effects of structural vacancies. The research into hexacyanometalates has thus focused on several key areas: novel material discovery, where computational tools and design principles are employed to identify new hexacyanometalates with optimized properties; the creation of synergistic hybrid materials that meld the strengths of both inorganic and organic components for superior performance; the development of advanced synthesis techniques tailored specifically for hexacyanometalates to achieve precise control over their structure, morphology, and doping levels; and a deep fundamental understanding of the mechanisms that contribute to rate limitations, voltage fade, and capacity degradation, which is crucial for guiding targeted material design and optimization strategies. Despite the need for further performance improvements, the recent advancements in alternative cathode materials showcase significant potential for their future commercialization. By addressing the existing challenges and pursuing the outlined research directions, these materials hold promise for unlocking the next generation of high-performance and sustainable energy storage solutions.

### 2.2. Anode Materials for SSSBs

SSSBs offer rapid charging capabilities surpassing their lithium-ion counterparts due to enhanced sodium-ion diffusion within the solid electrolyte [70]. However, the development of SSSBs faces significant challenges in optimizing anode materials, which currently suffer from limited capacity, unstable performance, and potential safety concerns [71]. This section analyzes the key requirements and limitations of SSSB anodes, exploring different reaction mechanisms and promising material classes toward future advancement.

(i) Key performance requirements for SSSB anodes

High specific capacity is paramount; anodes must store a substantial amount of charge per unit mass to provide energy densities that are competitive or superior to those of lithium-ion technologies. Rate capability is equally crucial, as it pertains to the anode’s ability to facilitate rapid charging and discharging cycles, a feature essential for applications requiring quick power delivery and recharge. Cycling stability ensures that the anode maintains its capacity and structural integrity over numerous charge–discharge cycles, reflecting the material’s durability and long-term usability. Initial Coulombic efficiency is another significant factor; this measures the efficiency with which electrons are transferred in electrochemical systems, aiming to minimize irreversible capacity loss during the first cycle, which is vital for maximizing the effective usability of the battery from its first use. Finally, the anode material should exhibit a low charge/discharge plateau at a potential that is sufficiently low to prevent the formation of dendrites, thereby enhancing the safety and energy efficiency. These dendrites, if formed, can pierce the battery separator and pose serious safety risks including short circuits and thermal runaway. The chemistry behind these requirements involves complex interactions between the electrode material’s crystal structure, electronic conductivity, and ion transport properties.

(ii) Reaction mechanisms

Reaction mechanisms and material approaches for SSSB anodes encompass a variety of strategies, each with its own set of benefits and challenges. Intercalation mechanisms, employed by carbon-based materials, such as graphite and hard carbons, typically offer moderate capacities and good cyclability; however, they often have lower theoretical capacities compared with other materials [76]. In contrast, non-metallic elements like phosphorus and nitrogen as well as their metal or metalloid compounds utilize conversion reactions that provide high theoretical capacities. However, these materials tend to suffer from limited cyclability due to significant volume changes and the complexity of their reaction pathways [77]. Alloying mechanisms, involving alloys of silicon, tin, germanium, and phosphorus with metals, also boast high theoretical capacities. However, they face challenges such as drastic volume expansion during sodiation, which can lead to mechanical degradation and capacity fade over time. Each of these approaches requires careful consideration of the material properties and reaction dynamics to optimize performance and durability in SSSB anodes.

(iii) Future research frontiers

Opportunities for SSSB anodes are expanding as advancements in materials science and engineering continue to unfold. Novel material discovery is a crucial area, where computational tools and innovative synthesis strategies are employed to identify and optimize new materials with superior structural and electrochemical properties. Another promising direction is the design of composite electrodes that integrate these novel materials with conductive matrices or nano-structured architectures. This approach not only enhances electronic conductivity, but also helps to mitigate volume changes during battery operation. Surface engineering is also pivotal, focusing on modifying the surface chemistry of anode materials to promote stable and reversible sodium ion insertion/extraction processes, crucial for improving the lifespan and efficiency of SSSBs. Additionally, electrolyte optimization plays a key role, with research aimed at developing electrolytes that not only offer high ionic conductivity, but also maintain stable interfaces with the anode material, thereby enhancing the overall battery performance and safety. Unlocking the full potential of SSSBs hinges on significant advancements in anode materials. Addressing the limitations of existing materials through targeted research efforts in novel material discovery, composite design, surface engineering, and electrolyte optimization will pave the way for high-performance SSSBs with superior energy density, rate capability, and cycling stability.

#### 2.2.1. Sodium Metal Anodes

Sodium metal stands out as a highly promising anode material for next-generation sodium-ion batteries due to its exceptional theoretical capacity, boasting a remarkable 1165 mAh g^−1^, and its notably low redox potential of −2.71 V versus the standard hydrogen electrode (SHE). This combination of high capacity and low potential translates to the potential to achieve batteries with significantly enhanced energy density. However, despite these compelling advantages, the practical deployment of sodium metal anodes is currently hampered by several substantial challenges, primarily centered around inherent safety hazards and the pervasive issue of dendrite formation during the repeated charge and discharge cycles. These limitations severely restrict the long-term stability and overall reliability of batteries employing sodium metal. This section delves into a comprehensive examination of the fundamental functionalities and critical limitations associated with sodium metal anodes while also exploring various innovative strategies and ongoing research efforts aimed at achieving stable, and crucially, dendrite-free operation, which is paramount for their successful integration into energy storage systems.

The functionalities and inherent challenges associated with sodium metal anodes encompass several pivotal areas that are absolutely critical to the successful advancement and widespread adoption of sodium-ion battery technologies. The fundamental processes of stripping (sodium dissolution during discharge) and plating (sodium deposition during charge) on sodium metal electrodes involve the continuous and reversible migration of Na^+^ ions between the electrode and the electrolyte. This dynamic process necessitates the use of a bare current collector that is not only chemically and electrochemically stable, but is also capable of facilitating these reversible electrochemical reactions uniformly across its surface, thereby preventing the heterogeneous nucleation and subsequent growth of detrimental dendrites [78]. Furthermore, the compatibility of the sodium metal anode with the chosen cathode material plays an equally crucial role in the overall battery performance and design. Depending on the specific electrochemical characteristics of the cathode material, different strategies regarding the anode might be necessary, such as employing pre-sodiated anodes or utilizing pure sodium metal directly [77]. To illustrate, sodium-containing cathode materials such as NaNi_0.5_Mn_0.5_O_2_ can intrinsically supply the necessary Na ions via deintercalation during the initial charge. In contrast, Na-free materials like V_2_O_5_ or FeF_3_ require an external Na source—typically provided by a pre-sodiated anode or a Na metal counter electrode—to enable effective electrochemical cycling in full-cell configurations.

Safety represents another paramount concern that must be rigorously addressed for sodium metal anodes. The inherent high reactivity of sodium metal with both air and moisture necessitates the implementation of stringent handling and containment protocols throughout the entire battery assembly and operational lifespan. Failure to do so can lead to hazardous reactions, including the generation of flammable gases and potential thermal runaway events, underscoring the critical need for robust safety measures. Moreover, the persistent challenge of dendrite formation during the sodium deposition process poses a major obstacle to the practical viability of these anodes. These microscopic, needle-like structures can propagate through the electrolyte, eventually causing internal short circuits within the battery cell. Such short circuits can lead to rapid heat generation, potentially triggering thermal runaway and catastrophic battery failure, thus highlighting the absolute necessity for effective dendrite mitigation strategies to ensure long-term performance, and most importantly, the safety of sodium-based battery systems.

The strategies currently being explored and developed for achieving dendrite-free cycling in sodium metal anodes encompass a diverse range of innovative approaches, all meticulously designed to enhance both the safety and overall electrochemical efficiency of sodium-based batteries. One particularly effective strategy involves the judicious use of electrolyte additives, such as 1,3-diallyl imidazolium perchlorate, which has demonstrated the ability to promote a more uniform and smooth sodium deposition morphology, thereby effectively suppressing the nucleation and subsequent growth of dendrites [76,78]. In addition to electrolyte modifications, surface coating techniques, such as the application of a thin and uniform NaBr layer onto the surface of the sodium metal, create a protective interfacial barrier. This barrier helps to prevent uneven sodium deposition during plating and encourages a more planar growth mode, thus inhibiting dendrite formation and consequently extending the operational lifespan of the battery [76,79]. Engineering the solid electrolyte interphase (SEI), which forms naturally on the anode surface due to electrolyte decomposition, through various chemical and electrochemical treatments is another critical approach. By enhancing the stability, uniformity, and ionic conductivity of the SEI layer, it can facilitate a more homogeneous sodium deposition process and effectively inhibit the propagation of dendrites [76]. Furthermore, the design of the current collector itself, including careful consideration of its microstructural features and surface chemical properties, can significantly influence the initial nucleation and subsequent deposition behavior of sodium metal, potentially leading to a more dendrite-free operation and ultimately improving the overall electrochemical performance of the battery.

In conclusion, while sodium metal anodes undeniably offer immense potential for realizing high-performance sodium-ion batteries with superior energy densities, effectively overcoming the significant challenges associated with safety and the persistent issue of dendrite growth is absolutely critical for their successful and widespread practical implementation. Continued and focused research efforts directed toward the development of advanced electrolyte formulations, innovative surface modification techniques, and optimized current collector designs hold immense promise for unlocking the full potential of sodium metal anodes, ultimately realizing a safe, reliable, and high-energy density sodium-based battery technology for the future.

#### 2.2.2. Carbon-Based Anode Materials

Carbon materials are attractive Na-ion battery anodes due to their abundance, low cost, and structural diversity. This section covers non-graphitic and graphitic forms, noting their potential and limitations.

(i) Non-graphitic carbons

Hard Carbon:

Disordered turbostratic structures with large interlayer spacing efficiently accommodate Na^+^ ions, making them ideal for Na-ion batteries due to low cost, ~0.3 V vs. SHE low-voltage plateaus, and high theoretical capacity (~300 mAh g^−1^). Na^+^ storage follows a “card-house model” involving intercalation and pore filling. A sustainable and cost-effective preparation uses precursors like sucrose and biomass. Microstructure defects enhance Na+ diffusion and cycling stability.

**Figure 3:**
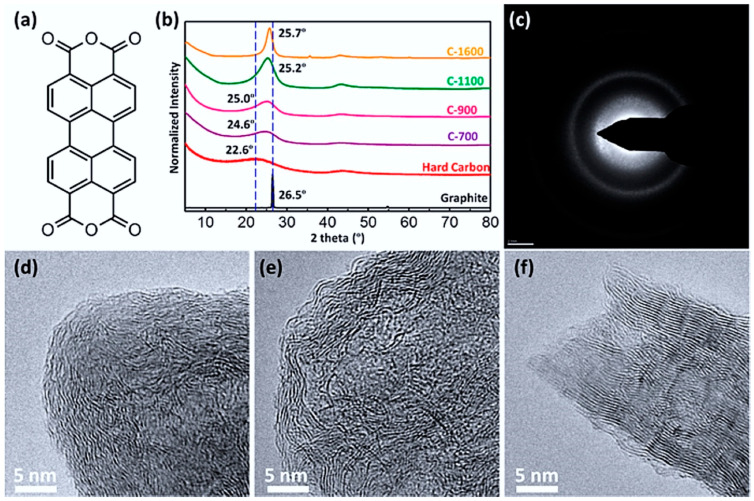
Soft carbon PTCDA [72]. (**a**) PTCDA molecular structure. (**b**) X-ray diffraction patterns of PTCDA-based soft carbons, hard carbon, and graphite (provides crystallinity information). (**c**) Selected-area diffraction image of C-900 (the existence of polycrystalline graphitic structures is suggested by the diffraction rings). (**d**) High-resolution transmission electron transmission image of C-900. (**e**) High-resolution transmission electron transmission image of C-1100. (**f**) High-resolution transmission electron transmission image of C-1600 (an ordered graphite structure appears within lattice fringes).

Soft carbon anodes:

Higher crystallinity with graphitic and disordered regions balances conductivity and capacity [72]. They offer exceptional rate performance and intrinsic dendrite-free operation above 0.2 V vs. SHE. Examples include carbon black and pitch-derived soft carbon (Figure 3), offering varied energy density and stability. Pyrolysis temperature can tune interlayer spacing for optimized performance [80].

(ii) Emerging carbon options

Flexible carbon cloth anodes:

Cost-effective due to fabrication from commercial cloth, offering mechanical flexibility and tailorable oxidation/conductivity. Ideal for diverse battery configurations and wearable tech. They can act as both the anode and substrate for Na_3_V_2_(PO_4_)_2_O_2_F cathodes in quasi-solid-state batteries, simplifying structure and reducing weight/volume [81,82].

Reduced graphene oxide (rGO):

Demonstrates multifaceted anode development via various production techniques. Valued for minimal volume changes during cycling, leading to extended cycle life, high-rate capability, and robust performance, achieving up to 300 mAh g^−1^ after 200 cycles [83]. Tunable defect densities and interlayer spacing optimize performance, highlighting the versatility of carbon-based materials for Na-ion battery anodes. Strategic tailoring techniques are crucial for enhancing Na-ion battery performance and implementation.

### 2.3. Electrolytes for Sodium-Ion Solid-State Batteries

SSSBs offer immense potential as a next-generation energy storage solution due to their inherent safety, improved cyclability, and fast charging capabilities [73]. However, realizing this potential hinges on resolving key challenges related to the electrolyte material and associated interfaces. This section delves into the limitations of the SEI and explores diverse electrolyte types, highlighting their advantages and research directions for improved SSSB performance.

(i) The solid-electrolyte interphase

The challenges associated with the SEI in SSSBs significantly affect the overall battery performance and its long-term cycling stability. One of the primary issues is the instability of the SEI layer, particularly when paired with high-voltage cathodes. This instability can lead to the dissolution of the SEI layer, triggering continuous side reactions that result in irreversible capacity loss and electrolyte depletion. Such degradation processes are detrimental to the longevity and efficiency of SSSBs. Additionally, the development of flexible electrodes that exhibit both excellent electrochemical performance and compatibility with these high-voltage environments presents another significant challenge. The design of these electrodes requires careful consideration of their mechanical and electrochemical properties to ensure that they are paired with safe, reliable electrolytes that support their functionality and meet specific performance criteria. These challenges highlight the need for innovative solutions in materials science and electrochemistry to optimize both SEI stability and integration of flexible electrodes in advanced battery architectures.

(ii) Electrolyte types and opportunities

Inorganic solid electrolytes:

These are pivotal in the advancement of sodium-ion batteries due to their high ionic conductivity and exceptional thermal stability, which are essential for high-performance energy storage systems. However, their widespread adoption is hampered by significant drawbacks such as limited mechanical flexibility and the inherent brittleness of these materials, which can pose safety risks under mechanical stress or thermal shock. To address these challenges, recent research has been directed toward the development of composite electrolytes. These innovative materials combine inorganic fillers with polymer matrices, creating hybrid systems that maintain the high ionic conductivity of traditional inorganic electrolytes while enhancing the mechanical flexibility and reducing safety concerns. Such advancements are crucial for improving the durability and safety of sodium-ion batteries, making them more viable for practical applications in various technologies. This approach not only mitigates the risks associated with inorganic solid electrolytes, but also opens new pathways for their integration into next-generation energy storage solutions.

Composite polymer electrolytes:

These ingeniously merge the beneficial properties of polymers and inorganic fillers, creating a synergistic effect that enhances the overall performance of the batteries. These electrolytes stand out by offering increased flexibility and processability, which are critical for practical battery manufacturing and integration into flexible device architectures. Additionally, they improve interfacial compatibility between the electrolyte and electrodes compared with their purely inorganic counterparts, potentially reducing interfacial resistance and enhancing ion transport. However, the development of composite polymer electrolytes also presents significant challenges. Ensuring adequate ionic conductivity while maintaining mechanical integrity is a delicate balance, as the inclusion of inorganic fillers can impact the polymer matrix’s viscoelastic properties. Furthermore, achieving long-term electrochemical stability remains a critical hurdle, as both polymer degradation and filler leaching can adversely affect battery life and performance. Addressing these challenges is crucial for advancing the technology toward commercial viability, requiring a deep understanding of both polymer chemistry and ceramic engineering to optimize the structure and composition of the electrolytes.

Solid polymer electrolytes:

These are characterized for their flexibility and inherent safety, which make them especially suitable for integrating with flexible electrodes. Unlike their inorganic counterparts, however, they generally exhibit a lower ionic conductivity, which can significantly limit the rate capability and overall energy density of the battery. To address these limitations, ongoing research is vigorously focused on enhancing the ionic conductivity of these electrolytes by incorporating conductive additives and innovatively tailoring the molecular structure of the polymers. These modifications aim to simultaneously optimize ion transport pathways and enhance the electrolyte’s mechanical properties. Additionally, overcoming challenges related to the SEI and designing electrolytes that are compatible with flexible electrode architectures are pivotal for the advancement of sodium-ion battery technology. By continuously exploring and innovating within the realms of composite and solid polymer electrolytes as well as advancing interface engineering techniques, researchers are paving the way toward developing stable, high-performance Na-ion batteries for a range of future energy storage applications.

#### 2.3.1. Inorganic Solid Electrolytes

Inorganic solid electrolytes (ISEs) have garnered significant interest for their role in SSSBs, positioning themselves as frontrunners to replace conventional liquid electrolytes. These electrolytes are distinguished by their superior ionic conductivity and excellent thermal stability, which could potentially enhance the safety and energy density of SSSBs. The exploration of ISEs is driven by their ability to sidestep the typical drawbacks of liquid electrolytes such as leakage risks, volatility, and thermal instability. However, challenges remain, particularly in the realms of mechanical flexibility and electrolyte–electrode interface stability, which are crucial for the practical application of SSSBs. This section delves deeper into both the promising attributes and the hurdles faced by ISEs, shedding light on how they influence the performance of SSSBs and highlighting the critical areas of future research needed to fully capitalize on these advanced materials in next-generation energy storage systems.

(i) Advantages of ISEs

ISEs bring several critical advantages to SSSBs that make them standout candidates in advanced battery technology. Notably, ISEs exhibit remarkable thermal stability, maintaining operational integrity across a broad temperature range from −50 °C to 200 °C. This capability is significantly superior to that of liquid electrolytes, which are prone to freezing and boiling under such extreme conditions [84]. This thermal resilience opens up new possibilities for deploying SSSBs in various climatic environments where traditional batteries might fail. Moreover, ISEs are characterized by their low activation energy, which promotes high ionic conductivity. This feature ensures that the conductivity remains stable despite temperature fluctuations, facilitating reliable battery performance across diverse operating conditions. The structure of ISEs, with their immobile anionic framework, is also crucial in minimizing bulk polarization. This structural advantage allows for higher power outputs and quicker charging times compared with traditional liquid electrolytes, where ion mobility can introduce significant resistance and slower charge rates [85]. Additionally, the use of ISEs enhances the energy density of SSSBs. Their solid, non-leaking nature allows battery cells to be packed closely together without the risk of electrolyte leakage, which is a common issue in batteries with liquid electrolytes. By eliminating the space typically required between cells to accommodate and contain liquid electrolytes, ISEs enable a more compact and efficient cell arrangement. This reduction in intercellular space maximizes the overall energy density of the battery system, making it possible to develop smaller, yet more powerful battery packs [86].

(ii) Challenges and limitations of ISEs

ISEs offer transformative advantages for SSSBs but they also present significant challenges that complicate their widespread adoption. One of the primary concerns is their inherent brittleness and limited mechanical stability. The rigid structure of ISEs can lead to difficulties during electrode integration and cell assembly, potentially resulting in performance degradation under mechanical stress or impact. This necessitates the development of novel cell designs that can accommodate the brittle nature of ISEs without compromising the battery’s structural integrity. Another critical challenge is ensuring interfacial stability between the ISEs and electrodes. Stable interfaces are crucial for maintaining low interfacial resistance and preventing capacity loss during repeated cycling. Incompatibilities at this interface can lead to increased resistance and degraded performance over time, posing a significant hurdle in the design of efficient battery systems. Additionally, while the ionic conductivity of ISEs is typically higher than that of traditional liquid electrolytes, it still falls short of the ideal values required for optimal battery operation. This lower ionic conductivity can limit the charging and discharging rates of SSSBs, affecting their overall efficiency and effectiveness. Enhancing the ionic conductivity of ISEs through materials engineering and advanced fabrication techniques remains a pivotal area of research, aimed at unlocking the full potential of solid-state battery technology.

#### 2.3.2. Composite Polymer Electrolytes

Composite polymer electrolytes for sodium-ion solid-state batteries represent a significant advancement in battery technology, blending the flexibility and processability of polymers with the enhanced ionic conductivity provided by inorganic components. As shown in Table 3, they can help mitigate high charge-transfer resistance at electrode–electrolyte interfaces and limitations in ionic conductivity. These electrolytes are crafted by embedding inorganic fillers into a polymer matrix, which not only improves the mechanical properties but also boosts ionic conductivity. Such a combination facilitates easier manufacturing and integration into battery systems while maintaining the safety profiles essential for consumer electronics, electric vehicles, and large-scale energy storage systems. The hybrid nature of the electrolyte structure allows them to bridge the gap between solid-state and traditional liquid electrolyte systems, offering a balance of good electrochemical stability and sufficient ionic mobility to support efficient charge transport. Research continues to optimize these materials to overcome challenges such as interfacial resistance and to achieve long-term cycle stability, which are crucial for the commercial viability of sodium-ion solid-state batteries. This section explores recent advancements in nanostructured materials and composite electrolytes, highlighting their potential to address these critical bottlenecks and pave the way for high-performance solid-state batteries.

(i) Interfacial challenges and solutions

Interfacial challenges in composite polymer electrolytes for sodium-ion solid-state batteries are largely due to the high charge-transfer resistance, a result of mismatched electronic and ionic conductivities between electrodes and solid electrolytes. This mismatch significantly hinders efficient charge transfer and limits the overall battery performance. To mitigate this, utilizing electrodes engineered with nanostructured features, such as nanoparticles or porous architectures, can substantially increase the interfacial contact area. This enhancement in contact area promotes improved charge transport, effectively reducing resistance [87]. Moreover, traditional solid electrolytes often showcase a lower ionic conductivity compared with liquid alternatives, impacting the battery’s rate capability and energy density. Addressing this limitation involves developing novel solid electrolyte materials that feature high intrinsic ionic conductivity and are structurally optimized to facilitate ion movement [87]. The use of composite electrolytes, which blend inorganic fillers with polymer matrices, leverages the high ionic conductivity of inorganic domains and the flexibility and processability of polymers to boost conductivity and interfacial compatibility [88]. In these composites, ionically conductive fillers such as Li-stuffed garnets actively contribute to enhanced ion transport, significantly increasing the electrolyte’s overall conductivity. On the other hand, passive fillers like oxide ceramics, although not contributing directly to ion transport, are instrumental in improving mechanical stability, suppressing dendrite growth, and improving the manufacturability of the electrolytes, which collectively lead to the development of higher-performing batteries.

**Table 3:** Comparison of composite polymer electrolytes for SSSBs.

Systems	Fillers	Electrochemical Stability Window/V (vs. Na^+^/Na)	Ionic Conductivity	Ionic Transference Number	References
PMA/PEG-NaClO_4_	Al_2_O_3_	4.5	1.46 × 10^−4^ at 70 °C	-	[75]
PEO-NaClO_4_	NaAlO_2_	4.5	7.4 × 10^−5^ at 30 °C	0.6	[89]
PEO-NaClO_4_	TiO_2_	-	2.62 × 10^−4^ at 60 °C	-	[20]
PVDF-HFP-NaTf	NZSP	5.0	1.2 × 10^−4^ at 0 °C	0.92	[75]
PEO-SN/PAN-NaClO_4_	NZSP	4.8	1.36 × 10^−4^ at 25 °C	0.42	[90]

#### 2.3.3. Solid Polymer Electrolytes

SPEs are emerging as a pivotal technology for sodium-ion solid-state batteries, primarily due to their remarkable safety features, inherent flexibility, and manufacturing ease. SPEs eliminate the risks associated with liquid electrolytes, such as leakage and flammability, thereby enhancing the overall safety profile of batteries. Their flexibility is particularly advantageous in applications requiring batteries to conform to unique shapes or withstand mechanical stress such as in wearable devices or flexible electronics. Additionally, SPEs can be processed at relatively low temperatures and with less stringent environmental controls compared with their inorganic counterparts, making them more adaptable to various fabrication techniques. This section describes the critical attributes of SPEs, detailing how their unique material properties contribute to improving battery performance by offering enhanced ionic conductivity, stability, and interface compatibility, all of which are essential for the efficiency and longevity of sodium-ion solid-state batteries.

(i) Composition and key properties

SPEs for sodium-ion solid-state batteries are designed to optimize battery performance through advanced material composition and structural innovation. These electrolytes primarily consist of sodium salts embedded in various polymer matrices, creating intricate supramolecular or macromolecular structures that facilitate the effective movement of sodium ions, essential for battery functionality.

Sodium salt selection:

Selecting the right sodium salts is pivotal as their chemical configuration significantly impacts the ionic conductivity of the electrolyte, and by extension, the overall electrochemical performance of the battery. Salts such as NaFSI (sodium bis(fluorosulfonyl)imide), NaTFSI (sodium bis(trifluoromethylsulfonyl)imide), NaDFOB (sodium difluoro(oxalate)borate), and NaClO_4_ (sodium perchlorate) are favored choices due to their excellent solubility in polymer matrices, which maximizes the availability of mobile sodium ions [80].

Polymer matrix attributes:

The selection of a polymer matrix plays a key role in enabling smooth and efficient ion movement inside the battery, primarily due to its ability to dissolve sodium salts and support ion transport. When sodium ions are able to dissociate easily and interact with the polymer chains, their movement across the electrolyte becomes more fluid and consistent. This ion mobility is essential for ensuring stable charge and discharge behavior. A good matrix helps maintain consistent ionic conductivity during cycling, which supports the battery’s efficiency and responsiveness over time. 

Polymer matrices are also chosen for their mechanical strength, flexibility, and ability to maintain structural integrity under operating conditions. They serve as a physical backbone that holds the electrolyte layer in place and prevents material breakdown over many cycles. This structural support is crucial for preserving contact between the active materials and current collectors, and for avoiding deformation, delamination, or internal shorting. The polymer must withstand thermal and mechanical stress while also enabling uniform ion flow. Altogether, these properties make the polymer matrix a critical component in ensuring long-term performance, safety, and reliability in sodium-ion battery systems.

Solvation process:

The solvation process relies heavily on the chemical affinity of the polymer matrix for sodium ions. Polymers with ether or oxygen-rich groups, such as poly(ethylene oxide) (PEO) and its derivatives, are particularly effective due to their ability to coordinate with sodium ions, thereby reducing the ion’s desolvation energy and promoting faster ion transport. This coordination transforms the rigid crystal structure of sodium salts into a more fluid environment that allows the ions to move freely, significantly reducing the energy barrier for ionic migration. Strategies for modifying the polymer backbone include the incorporation of side groups that enhance flexibility and ionic conductivity. Cross-linking within the polymer structure or the use of plasticizers can also improve flexibility and ionic mobility, albeit sometimes at the cost of reduced mechanical strength [81].

Exceptional mechanical strength:

The mechanical integrity of SPEs is crucial for withstanding the physical stresses of operation and preventing the formation of detrimental sodium dendrites. These dendrites can breach the electrolyte layer, leading to short circuits and potential battery failures. Polymers selected for SPEs often exhibit high tensile strength and elasticity, providing the necessary robustness to combat mechanical stresses and maintain structural integrity under operational conditions. Amongst the promising strategies for designing stable solid polymer electrolyte membranes is hot-pressing to realize high-energy-density sodium metal batteries. Overall, the polymer matrix must possess sufficient mechanical strength to maintain structural integrity and protect against mechanical degradation or puncture [82].

High bond flexibility:

The flexibility of the polymer bonds plays a vital role in ion transport. Polymers that exhibit high segmental mobility facilitate easier and more efficient ion migration by lowering the energy barrier associated with ion movement. This flexibility can be enhanced by using polymers with inherently flexible backbones or by adding plasticizers to the polymer matrix, thus improving the overall ionic conductivity and performance of the electrolyte. Overall, the polymer’s flexibility can impact the electrolyte’s ability to accommodate volume changes during ion insertion and extraction without cracking or degrading. Ultimately, the design of high-voltage SPEs requires the design optimization of the structure of the polymer matrix, the molecular interactions within the electrolyte, and the interface architecture between the electrode and electrolyte [83]. All of these polymer attributes—solvation capability, mechanical strength, and flexibility—must be finely balanced to enhance the functionality of SPEs. This balance between ionic conductivity and mechanical integrity is crucial for developing efficient, durable, and safe sodium-ion solid-state batteries that offer high performance and reliability.

(ii) Advantages and performance

SPEs bring a suite of advantages that make them particularly attractive for modern battery manufacturing technology. These electrolytes are non-flammable, significantly enhancing safety by eliminating the risks associated with conventional liquid electrolytes that contain volatile components. This safety feature is critical for applications where battery failure poses significant risks such as in electric vehicles and portable electronic devices. Furthermore, SPEs exhibit exceptional flexibility, which allows them to conform to a variety of electrode structures. This adaptability is crucial for developing advanced, bendable energy storage devices that can be integrated into next-generation flexible electronics. Additionally, the processability of SPEs stands out; they can be efficiently produced through scalable and cost-effective methods like solution casting. This manufacturing versatility facilitates the integration of SPEs into commercial production lines, enhancing the feasibility of transitioning to solid-state battery systems in industrial applications. Collectively, these factors—safety, flexibility, and processability—position SPEs as pivotal components in the evolution of safer, more adaptable, and economically viable sodium-ion solid-state batteries. Various types of SPE and their comparison can be found in Table 4. As an example, a particularly promising SPE for sodium-ion solid-state batteries is composed of NaFNFSI (sodium (fluorosulfonyl)(n-nonafluorobutanesulfonyl)imide) dissolved in a PEO (poly(ethylene oxide)) matrix [77]. Utilizing a casting method for fabrication, this electrolyte exhibits notable performance characteristics including a high ionic conductivity of 3.36 × 10^−4^ S cm^−1^ at 80 °C, excellent electrochemical stability with an anodic window exceeding 4.87 V, impressive thermal stability above 300 °C, and remarkable cycling performance, demonstrating 70% capacity retention after 150 cycles at 1 °C [23]. These combined attributes suggest the significant potential of NaFNFSI-PEO SPEs for enabling high-performance, safe, and durable SSSBs.

In sodium-ion battery systems, a critical trade-off exists between the ionic conductivity and mechanical properties of SSEs: materials engineered for high ionic conductivity (e.g., fast ion movement through less hindered pathways) often lack the mechanical strength and flexibility needed to withstand electrode volume changes and suppress dendrite growth, while mechanically robust SSEs (e.g., rigid, crystalline structures) can suffer from lower ion mobility and poor interfacial contact. For instance, highly conductive inorganic SSEs like NASICON are typically brittle, whereas flexible polymer electrolytes, while offering good interfacial contact, often have lower room-temperature ionic conductivity. This dilemma is frequently addressed through composite solid-state electrolytes, where inorganic fillers are incorporated into a polymer matrix. The optimal ratio of these fillers is crucial; for example, adding a relatively low content of inorganic fillers, such as 5 wt.% of a modified MOF, to a PEO/NaClO_4_ polymer can significantly boost the ionic conductivity by disrupting polymer crystallization and creating new conductive pathways, while a higher filler content might prioritize mechanical robustness at the expense of processability or flexibility, as seen in some polymer-in-ceramic systems where the ceramic component can reach 75 wt.% [80,81,82].

Overall, SPEs offer a promising avenue for advancing sodium-ion solid-state batteries, given their unique combination of properties and performance. Continued research focused on optimized polymer design, advanced fabrication techniques, and tailored sodium salt selection holds immense potential for realizing high-performance batteries with enhanced safety, flexibility, and durability.

## 3. Other SSB Systems

K-ion batteries have experienced significant developments in recent years. Initially, after their introduction in 2004, research on K-ion batteries has faced stagnation due to safety concerns and the popularity of LIBs and Na-ion batteries [92]. Within the last decade, the interest in K-ion batteries has been rekindled with the exploration of reversible potassium insertion into graphite as anodes, leading to a surge in scientific publications and the investigation of various electrode materials [84]. Despite this progress, systematic research on the electrolyte and its impact on K-ion battery performance with regard to safe operation has been lacking. More recently, a breakthrough was made in the development of K-ion batteries with the synthesis of a hybrid electrolyte using commercial materials. This demonstrated suitability for low-temperature operation and showcased desirable properties such as ionic mobility, electrochemical stability, and mechanical flexibility [14]. Although challenges and limitations remain, the recent developments in electrode materials and electrolytes highlight the growing interest and potential for further progress in the field of K-ion batteries.

Mg-ion batteries have potential as substitutes for LIBs, but developing suitable electrolytes has been a major challenge. SSEs offer stability, but their low conductivity at room temperature hinders magnesium ion movement. Various SSEs, including ceramic, polymer-based, and composite electrolytes, have been developed to enable magnesium ion conduction. Ceramic-based electrolytes, for instance, aim to enhance ionic conductivity through structural modifications. In 2017, trinary spinel chalcogenides were discovered, which showed high magnesium mobility by placing magnesium in a tetrahedral site with an expanded volume per anion. Subsequent studies have focused on SPEs with varying conductivities, where improvements have been made by adding plasticizers or using solvating blend polymers. These innovations yielded promising electrochemical performance including high ionic conductivity and stability. For example, in 2020, a porous polymer electrolyte demonstrated reversible magnesium ion plating/stripping, outperforming traditional liquid electrolytes. Composite electrolytes, which combine the advantages of polymers and ceramics, have shown improved ionic conductivity, elevated transference numbers, and compatibility with electrodes. Around the same time frame, another study reported on a rechargeable SSE Mg-ion battery operating at room temperature. Overall, the development of Mg-ion batteries requires compatible electrolytes. SSEs, including ceramics, polymers, and composites, enhance magnesium ion conduction. Recent advances have brought Mg-ion batteries closer to practical applications, as evidenced by the recent increase in publications and patenting activity.

### 3.1. Solid-State K-Ion Batteries (SSKBs)

**Figure 4:**
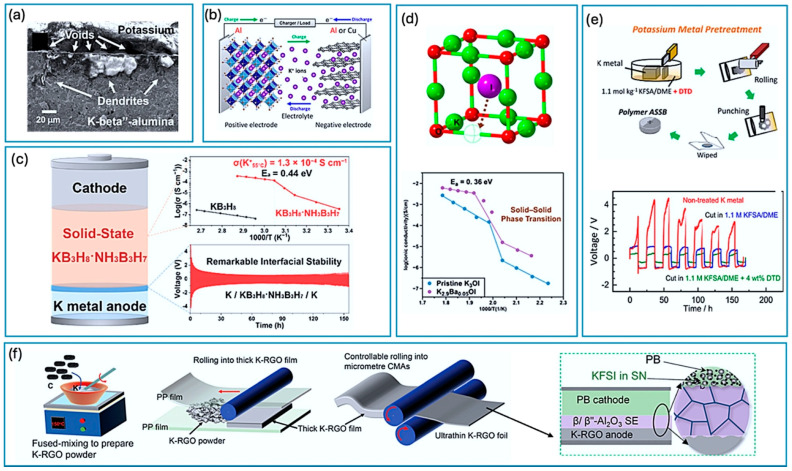
(**a**) Scanning electron micrograph demonstrating the K/K-beta″-alumina interface after cycling caused voids and dendrite formation [100]. (**b**) Schematic of the K-ion battery demonstrating K^+^ movement. Reprinted with permission. Copyright 2020, American Chemical Society [85]. (**c**) Schematic of SSKBs with the KB3H8 ·NH_3_B_3_H_7_ solid electrolyte. The ionic conductivity of the electrolyte is graphed compared with KB_3_H_8_, and the voltage across the anode/electrolyte interphase is also depicted. Reprinted with permission. Copyright 2022, American Chemical Society [88]. (**d**) Body-centered cubic structure of K_3_OI with the graph demonstrating the ionic conductivity of doped and pristine K_3_OI. Reprinted with permission. Copyright 2021, American Chemical Society [99]. (**e**) Pretreatment process for K metal anodes and stripping–deposition tests (4.3 μA cm^–2^ at 25 °C) of the treated and untreated K/SPE/K cells [93]. (**f**) Schematic demonstrating the fused-rolling process of the K-RGO anode and resulting battery cell. Reprinted with permission. Copyright 2023, Wiley [104].

In addition to Na-ion batteries, K-ion batteries have recently received much attention due to the inexhaustible abundance and cost-effectiveness of potassium compared with Li [85]. Additionally, SSKBs are also auspicious due to their high-power density, high operational voltages, and safety. Unfortunately, like many other SSBs, K-ion based batteries currently face many struggles including interfacial issues at the electrode/electrolyte interfaces for both potassium- and carbon-based electrodes. This includes issues with dendrite formation at K anodes (Figure 4a), interfacial resistance, and a lack of theoretical models of SEIs [86,87,88,93]. For SSKBs, solid electrolyte issues arise such as low ionic conductivities, low energy density, and costly fabrication methods, although solid electrolytes are proving to be better than liquid electrolytes [92]. There are several avenues toward fixing interfacial issues in SSKBs, some of which will be discussed here along with recent general information regarding the current state of SSKBs.

Many types of electrolytes (i.e., organic, inorganics, and hybrids) responsible for ion transfer between electrodes (Figure 4b) have been investigated for SSKBs, with the most promising candidates being polymer and inorganic electrolytes [92]. The use of polymer electrolytes for SSKBs understandably share many of the same advantages and disadvantages as polymer electrolytes for the other SSBs described in previous sections. Namely, polymer electrolytes are promising due to their flexibility and safety but can exhibit low ionic conductivity and are temperature dependent [86]. Research focusing on tailoring novel polymer electrolytes for K-ion based batteries will help overcome current issues including interfacial resistance. As previously discussed, further investigation of promising polymer electrolytes in other SSB systems, particularly PVDF-HFP, PMMA, and PAN based electrolytes, should be studied for applications in SSKBs [94]. Second, additives (e.g., fluoroethylene carbonate) should be considered to overcome small electrochemical potential windows and poor ionic conductivity seen in some polymer electrolytes via either theoretical, simulation, or experimental studies [86].

Solid electrolyte and electrode interface issues may be solved by thoughtfully crafting the solid electrolyte. For example, the recently investigated solid electrolyte for SSKBs, KB_3_H_8_·NH_3_B_3_H_7_, is promising [88]. The electrolyte was crafted by mechanically ball-milling KB_3_H_8_ and NH_3_B_3_H_7_ under an argon atmosphere and pressing the powder into pellets. Pellets were then assembled into an SSB with a K anode and PTCDA cathode. When tested, the K/KB_3_H_8_·NH_3_B_3_H_7_/PTCDA SSB demonstrated good interfacial stability with the K anode and the highest K-ion conductivity reported (1.3 × 10^–4^ S cm^–1^ at 55 °C) within the range of Li-ion batteries (10^−3^–10^−8^ S cm^−1^) (Figure 4c) [95]. This is advantageous as interface issues pose a major barrier for SSBs, and additionally, SSBs need a high ionic conductivity to be effective [96,97]. Despite promising results, such as the high activation energy (0.44 eV), the K/KB_3_H_8_·NH_3_B_3_H_7_/PTCDA SSB demonstrated poor cycling behavior as it only worked for about 45 cycles at a discharge capacity ranging from 49.1 to 77.4 mAh g^−1^. This was thought to be the result of the failures of the PTCDA interface, as the K anode demonstrated more promising behavior and dendrite suppression despite the reactivity of K [88,92]. Ultimately, while this electrolyte shows promise, the synthesis methodology may present a new problem, as the high stack pressure required to craft pressed-pellet type cells are not ideal for future commercial production [97].

Antiperovskite electrolytes are also promising for SSBs, including SSKBs, because of their flexible and tunable structure [98]. Specifically, body-centered cubic (bcc) antiperovskites are promising, as the structure contains many channels that allow for fast ion migration [99]. Because of the benefits of the bcc structure, K_3_OI has emerged as a promising solid electrolyte for SSKBs (Figure 4d). The K_3_OI electrolyte was fabricated and formed into pellets via hot pressing (400 MPa), then sintered at 220 °C for 8 h without repeated grinding and heating or high-energy ball milling. After doping the electrolyte with Ba^+^, the electrolyte showed a higher ionic conductivity of 3.5 × 10^−3^ S cm^–2^ and a low activation energy of 0.36 eV (Figure 4d). When the electrolyte was constructed into a battery cell with K and carbon paper electrodes, the cell was cycled at 270 °C: at a current density of 0.2 and 0.5 mA cm^−2^, the overpotential was approximately 20–50 mV [99]. The need to operate the battery at high temperatures is not an uncommon issue with SSKBs and hinders many solid electrolytes [92]. The other solid electrolytes mentioned within this review demonstrate a better alternative for operation at lower operating temperatures.

The dendrite suppression at the K anode noted in the aforementioned study was further investigated to determine why dendrites and voiding plague SSKBs to a lesser extent than Li or Na SSBs [100]. Specifically, SSKBs, such as K anode solid-state cells with a K-beta″-alumina ceramic electrolyte, function with higher critical currents for dendrite growth (4.8 mA cm^−2^) than Li and Na metal anode SSBs. The critical current was determined by cycling the cell at increasing current densities (0.05, 0.1, 0.2, 0.3, 0.4, 0.5 mA cm^−2^) until failure, and was amongst the highest reported for SSBs [101,102]. It was determined that the mechanical properties of K were responsible for reduced interfacial issues at the K anode and solid electrolyte interface. Specifically, the low yield strength and readiness to creep under low pressures aided in the K anode’s ability to sustain high rates of plating and stripping without failure, which allowed for high charge and discharge rates [100].

Interfacial issues further plague emerging polymer electrolytes at the K electrode interface, and another avenue toward fixing interface issues could be pretreating the electrode. Hamada et al. pretreated the K metal electrode with a 1.1 mol·kg^−1^ KFSA/DME + DTD (potassium bis(fluorosulfonyl)amide; 1,2-dimethoxyethane; 1,3,2-dioxathiolane 2,2-dioxide, respectively) liquid electrolyte by dipping and cutting the metal while submerged in the solution (Figure 4e) [93]. When tested with a solid polymer electrolyte (SPE), the battery cell was found to be a 3 V-class all-solid-state K-ion battery at 25 °C. Additionally, the K/SPE/K cells had a current density of 3.9 mA g^−1^ and interfacial resistance of 2 × 10^5^ Ω cm^−2^. The study proposed that the addition of DTD helped to reduce the polarization of the cell and in turn better the interfacial resistance. The better interfacial resistance also allowed for a high reversible capacity of 220 mAh g^−1^ [93]. This pretreatment of the K anode was simple, effective, and helped to better the interfacial resistance issue of SSKBs that utilize polymer-based solid electrodes [92].

While K may intrinsically be better fit as an electrode material than other metals due to its mechanical properties and its reductive potential (−2.93 V vs. SHE), it does still demonstrate some dendrite formation, which needs to be addressed [100,103]. The answer may lie in composting K anodes with a carbonaceous material to increase the SE affinity and reduce interfacial resistance [92,103,104]. A recent study crafted an ultrathin (50 µm) composite metallic anode (K-10% reduced graphene oxide (RGO)) that demonstrated negligible interfacial resistance (1.3 Ω cm^2^) with K^+^ diffusion (2.37 × 10^−8^ cm^2^ s^−1^) in a K-RGO/β/β″-Al_2_O_3_/KxFe[Fe(CN_6_)] cell (Figure 4f). The anode was crafted by mixing reduced graphene oxide with molten K, and after the mixture cooled, the material was shaped into a uniform and tunable thickness by mechanical rolling (Figure 4f). Furthermore, the cell operated at −20–120 °C with a retention of 94.4% after 150 cycles, demonstrating cyclability and temperature stability (200 °C versus 63.4–150 °C for K) [104]. This battery and the others summarized here demonstrate promise for SSKBs with improvements in ion conductivity and interfacial resistance, which are current challenges for SSKBs.

### 3.2. Solid-State Mg-Ion Batteries (SSMBs)

Mg-based SSBs are another promising candidate to replace Li-ion batteries and have received increasingly more attention in recent years. Similar to K-based SSBs, Mg-ion SSBs are promising due to the natural abundance, low density, and high energy density of Mg [105]. Mg is further advantageous because of its high theoretical capacity (3833 mAh cm^−3^) due to the two electrons released in redox reactions, and because like many other Li alternatives, SSMBs are also considered to be safer than the current Li-ion batteries [106]. However, the low mobility of Mg ions within solids at room temperature, the limited reversibility, and poor cycling stability pose a problem for SSMBs [106,107,108]. Additionally, electrolytes for SSMBs further present functionality issues due to electrode incompatibilities, corrosiveness, and synthesis methodologies, warranting further study [107]. Many different types of solid electrolytes, summarized previously to this review (i.e., oxides, hydrides, chalcogenide, MOFs, polymers and composites), are currently being studied to better Mg-ion SSBs, and a few recent advancements in electrolytes are presented below in addition to the current electrode progress within the literature [106,108].

**Figure 5:**
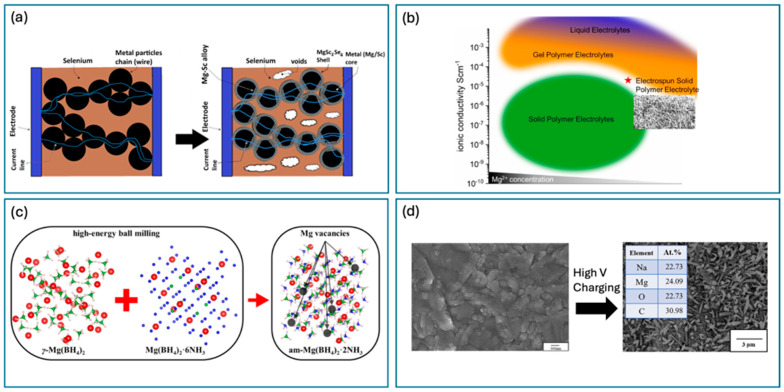
(**a**) Schematic of how the (Mg–Sc–Se) pellet changes after DC current flow. Reprinted with permission. Copyright 2022, Wiley [109]. (**b**) Ionic conductivity of electrospun PEO: Mg(TFSI)_2_ solid polymer electrolyte compared with other polymer Mg electrolytes [113]. (**c**) Schematic of m-Mg(BH_4_)_2_2NH_3_ fabrication to form Mg vacancies. Reprinted with permission. Copyright 2023, Wiley [107]. (**d**) Surface morphology Mg electrode before and after high-voltage charging [105].

The low mobility of Mg ions poses a problem for electrolytes in SSMBs and are being investigated to improve ion mobility [109]. Magnesium scandium chalcogenide spinels were suggested early on as an electrolyte solution demonstrating promise theoretically and experimentally due to the high Mg mobility granted by the spinel structure [109]. Advances in the synthesis methodologies of MgSc_2_Se_4_ (Figure 5a) are working to lower the electronic conductivity through room temperature electric field-assisted sintering of Mg, Sc, and Se powders into pellets and sandwiching between gold foil coated electrodes. Kundu et al. [109] reported that the MgSc_2_Se_4_ electrolyte had a desirably low electronic conductivity of 10^−11^ S cm^−1^ and an ionic conductivity of 1.78 × 10^−5^ S cm^−1^ due to the room temperature synthesis methods and the addition of extra selenium (Figure 5b). This level of ionic conductivity is within range of current Li-ion SSBs (10^−8^–10^−3^ S cm^−1^) and demonstrates promise for MgSc_2_Se_4_ as an electrolyte for SSMBs [95]. However, the study noted that the ratio of selenium in the electrolyte mixture needs to be balanced in order to achieve this lower electronic conductivity. Balancing this ratio can be difficult because the amount of selenium lost during the synthesis process requires an overestimation, however, an incorrectly high ratio of selenium will undesirably increase the electric conductivity. Kundu et al. further mentioned that the DC current application schedule during synthesis could be limiting in the large-scale production of the electrolyte. Despite the promising nature of MgSc_2_Se_4_ as a solid electrolyte for SSMBs, these intricacies of fabrication may warrant further study into solid electrolytes to craft functional SSMBs. Qian et al. [110] identified a novel structural motif in ABO_4_-type zircon materials that promoted high Mg-ion mobility, making them promising candidates for magnesium intercalation cathodes in multivalent batteries. Through computational predictions and experimental validation, the study demonstrated good Mg-ion transport properties in sol–gel synthesized zircon materials such as YVO_4_, EuVO_4_, and EuCrO_4_, with EuVO_4_ exhibiting the best electrochemical performance. This work introduced a new structural design metric for future Mg cathode development based on zircon materials.

Borohydrides are also promising electrolytes for SSMBs and demonstrate some of the higher ionic conductivities for SSMBs [108]. In particular, amorphous Mg borohydride ammoniate, Mg(BH_4_)_2_ ·2NH_3_, has emerged as a potential candidate as a solid electrolyte in Mg-ion SSBs [106,107]. The electrolyte was synthesized through 24 h of the high-energy ball milling of Mg(BH_4_)_2_ and Mg(BH_4_)_2_ ·6NH_3_ and pressing under a 100 MPa stack pressure to form pellets (diameter = 10 mm), after which Mg foil and TiS_2_ were utilized as electrodes for testing (Figure 5c). The electrolyte demonstrated promising Mg-ion conduction properties, namely, a transference number of 0.95 and a high ionic conductivity (5 × 10^−4^ S cm^−1^, 75 °C). This ionic conductivity is extremely promising, as it is higher than other SSMB electrolytes, including the magnesium scandium chalcogenide spinels discussed earlier, and is again within the range of current Li-ion SSBs [95,107,109]. This resultant high Mg-ion conduction was investigated through DFT, and Mg vacancy migration was identified as the main driver of the increased ion mobility, as the Mg vacancy formation energy was lower than the Mg interstitial formation energy (1.92 versus 2.21 eV, respectively), both of which contribute to ion conduction and are formed during synthesis. The amorphous structure, which was thoughtfully selected, was additionally thought to be responsible for the high ionic conductivity, as crystallinity is known to reduce ionic conductivity [110,111]. Additionally, Mg(BH_4_)_2_ ·2NH_3_ demonstrated an apparent electrochemical stability window of 0–1.4 V, and the Mg battery cell could be successfully cycled for 710 h at 0.05 mAcm^−2^ with an initial reversible specific capacity of 141 mAh g^−1^. The critical current density before dendrites caused a short circuit was agreeably found to be 3.2 mA cm^−2^ [106,107]. Overall, the ionic conductivity of Mg(BH_4_)_2_ ·2NH_3_ demonstrates promise, but other parameters, namely the electrochemical window, need to be considered and improved.

Despite the comparatively lower ionic conductivity of polymer-based electrolytes, polymer-based solid electrolytes demonstrate some promise for SSMBs. In particular, through the use of proven strategies, such as nanostructure engineering, the ionic conductivities of solid electrolytes can be improved [112]. Electrospinning is a prime example of nanostructure engineering in solid electrolytes that has demonstrated promise as a synthesis method for polymer-based solid electrolytes for SSMBs. Through the use of electrospinning techniques, the fabrication of large-scale fibrous material of solid electrolytes offers increased ion mobility and transport. Recently, the poly(ethyleneoxide)-based (PEO) solid polymer electrolyte Mg(TFSI)_2_:PEO was found to have a room temperature ionic conductivity of 2 × 10^−5^ S cm^−1^ (25 °C) (Figure 5c), as determined by impedance spectroscopy, which was found to be higher than those previously reported for similar samples formed by solution casting [113]. The reported ionic conductivity is an improvement over previous polymer-based solid electrolytes used for SSMBs, and is of the same magnitude as other SSMB solid electrolytes reported within this review.

**Figure 6:**
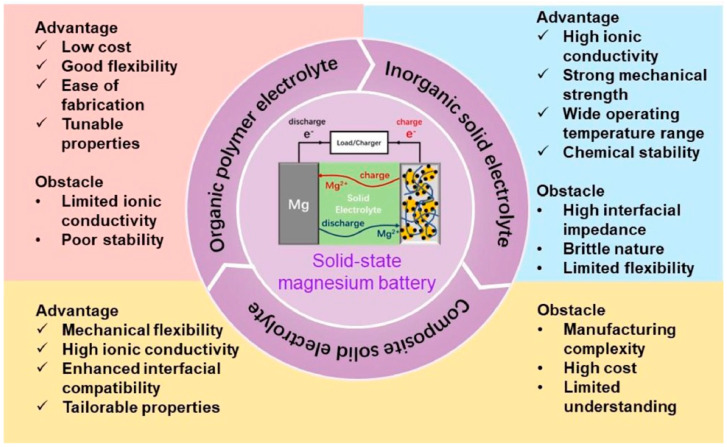
Advantages and disadvantages of electrolytes for solid-state magnesium batteries. Copyright 2024 [115].

Electrodes for SSMBs, in addition to solid electrolytes, also require further investigation before an effective SSMB can be crafted. Mg anodes are commonly used in SSMBs because the material presents the benefit of having a low electrode potential (−2.73 V) and is less prone to dendrite formation compared with other materials [105,106,113,114]. Currently, the fabrication of Mg anodes is principally focused on Mg coating methods, mainly powder-based and thin-film electrodes. This methodology results in electrodes with a low active material density that are easily oxidized, which limits their usability. Chen et al. recently sought to better the coating process through the use of radio frequency sputtering to deposit Mg onto a copper foil. The resultant Mg anode had a fair compactness and flatness (400 nm) that allowed for deterioration resistance and increased electrode/electrolyte contact area. When tested with a flexible Mg silicate electrolyte and carbon black cathode, the battery exhibited a desirable charge–discharge capacity due to the high interfacial adhesion [105]. However, a passivation layer formed during rapid charging, which was detrimental to the electrical function of the battery (Figure 5d). Further research into Mg electrodes cannot be neglected in the process of developing a better SSMB and warrants further investigation to better understand and prevent the formation of a passivation layer and other interfacial related issues. The advantages and disadvantages of electrolytes for solid-state magnesium batteries are summarized in Figure 6.

## 4. Present Challenges and Future Perspectives

### 4.1. Present Challenges

Solid-state battery systems using Na-ion, K-ion, and Mg-ion charge carriers offer the potential for enhanced safety and energy density (Table 5) compared with their liquid-electrolyte counterparts, particularly Li-ion batteries. However, significant challenges remain in their development and commercialization. Here, we present a discussion of some specific challenges for each system [116].

Na-ion batteries are attractive due to the abundance and lower cost of sodium compared with lithium. However, solid-state Na-ion systems face several hurdles:

Low ionic conductivity: Many current Na-ion solid electrolytes exhibit lower ionic conductivity at room temperature compared with liquid electrolytes and some Li-ion solid electrolytes [117]. This limits the power density and overall performance of the battery. Achieving sufficient Na-ion mobility within the solid electrolyte structure is a key challenge [118].

High interfacial resistance: Poor contact and high interfacial resistance between the solid electrolyte and the electrodes (anode and cathode) hinder efficient ion transport [119]. This leads to lower energy efficiency and power capability. Creating and maintaining stable, low-resistance interfaces is crucial [120].

Electrolyte–electrode compatibility: Chemical and electrochemical incompatibility between the solid electrolyte and electrode materials can lead to the formation of resistive interphases, capacity fade, and poor cycling stability. Identifying and developing compatible material combinations is essential [121].

Mechanical stability: Solid electrolytes, particularly ceramics, can be brittle and susceptible to cracking under mechanical stress during battery operation (e.g., volume changes in electrodes during cycling) [122]. Maintaining mechanical integrity and good interfacial contact over long cycles is challenging.

Dendrite suppression: While sodium is less prone to dendrite formation than lithium, it can still occur under certain conditions, especially with metallic sodium anodes. The solid electrolyte needs to effectively suppress any potential dendrite growth to ensure safety and a long cycle life [123].

Scalability and cost: Scaling up the production of high-performing Na-ion solid electrolytes and solid-state batteries at a competitive cost is a significant challenge for commercialization [124].

K-ion batteries are another promising alternative due to the high abundance and low cost of potassium. However, K-SSBs face even more pronounced challenges.

Very limited solid electrolytes: The development of solid electrolytes with high K-ion conductivity is still in its early stages. Fewer K-ion solid electrolytes with acceptable performance have been reported compared with Li-ion and even Na-ion systems [125].

Low ionic conductivity: Existing K-ion solid electrolytes generally exhibit a significantly lower ionic conductivity than their Li-ion and Na-ion counterparts, especially at room temperature [126]. This severely restricts their practical application.

Large ionic radius and slow kinetics: The larger ionic radius of K^+^ compared with Li^+^ and Na^+^ leads to sluggish intercalation kinetics in electrode materials and slower diffusion within the solid electrolyte [127].

Significant volume changes: Electrode materials for K-ion batteries often experience even larger volume changes during cycling than those in Na-ion or Li-ion batteries, posing significant challenges for maintaining mechanical stability and interfacial contact with the solid electrolyte [128].

Dendrite formation: Potassium is highly reactive, and dendrite formation with potassium metal anodes is a significant safety concern that needs to be effectively suppressed by the solid electrolyte [129].

Electrolyte–electrode compatibility: Similar to Na-ion systems, finding chemically and electrochemically stable interfaces between K-ion solid electrolytes and electrode materials is crucial but challenging [130].

Mg-ion batteries offer the potential for higher volumetric energy density due to the divalent nature of Mg^2+^. However, Mg-SSBs face the most significant technical barriers.

Sluggish Mg-ion conduction: The high charge density of Mg^2+^ leads to strong electrostatic interactions with the solid electrolyte lattice, resulting in very low Mg-ion mobility and poor ionic conductivity at room temperature for most reported solid electrolytes. This is a major bottleneck [131].

Divalent ion transport mechanism: The transport mechanism of divalent Mg^2+^ in solids is more complex than that of monovalent Li^+^ or Na^+^, often involving strong correlations and leading to high activation energies for migration [132].

Electrolyte–electrode compatibility issues: Many conventional electrolytes are incompatible with magnesium metal anodes, leading to the formation of passivating layers that hinder Mg-ion transport. Finding suitable electrolytes that allow for reversible Mg plating and stripping is a significant challenge [133]. This issue extends to solid electrolytes as well.

Cathode material limitations: The sluggish diffusion of Mg^2+^ ions in many potential cathode materials limits the rate capability and overall performance of Mg-ion batteries. Identifying and designing cathode materials with fast Mg-ion intercalation kinetics is crucial [134].

High interfacial resistance: Achieving good contact and low interfacial resistance between Mg-ion solid electrolytes and electrodes is particularly challenging due to the poor wetting and slow kinetics [135].

Absence of suitable SEI: Unlike Li-ion batteries where a stable SEI forms on the anode, a similar beneficial interphase has been difficult to achieve in Mg-ion systems, contributing to interface instability and capacity fade [136].

Dendrite suppression: While magnesium metal is less prone to dendrite formation compared with lithium, it is still a concern, and the solid electrolyte must prevent any potential growth [137].

There are even further prospects for solid-state Na-ion, K-ion, and Mg-ion batteries, where optimizing oxygen reduction/evolution reaction (ORR/OER) kinetics would be critical for high performance and cyclability. Specifically, ORR and OER would be central to the performance of future metal–air batteries (Na–air, K–air, and Mg–air), where oxygen participates directly in electrochemical reactions. In solid-state systems that involve conversion-type cathodes or hybrid air electrodes, sluggish oxygen reaction kinetics are a major bottleneck due to poor ion transport and limited active surface area. In general, solid–solid interfaces often have high charge-transfer resistance and limited reaction sites, leading to slow ORR/OER kinetics. Unlike liquid electrolytes, solid electrolytes lack a mobile oxygen reservoir, making catalytic enhancement strategies crucial. Future strategies for development and optimization would vary based on the specific chemistry and ion size. For Na-ion solid-state batteries, enhancing the ORR/OER kinetics can involve developing cathode materials with tailored electronic structures, such as introducing anionic redox or surface modifications with nanoscale catalysts (e.g., noble metals or single-atom catalysts), to lower the overpotentials [15,138,139,140,141]. Additionally, optimizing the solid electrolyte/cathode interface for improved ion transport and reduced interfacial resistance is crucial, potentially through the atomic layer deposition of thin protective layers or fabricating composite cathodes with intimately mixed ionically conductive phases. For K-ion systems, the larger ionic radius of K^+^ presents further challenges. Strategies could focus on designing open framework cathode structures with wider diffusion channels and stable architectures to accommodate K^+^ insertion/extraction without significant volume changes that can impede ORR/OER. Furthermore, surface engineering with highly conductive coatings or integrating catalysts that specifically promote facile oxygen redox pathways in the presence of larger K^+^ ions would be paramount. This might involve doping the cathode surface with elements that act as Lewis acid/base sites to stabilize reaction intermediates [142,143,144,145,146]. For Mg-ion solid-state batteries, the sluggish kinetics due to the divalent nature of Mg^2+^ (higher charge density leading to stronger electrostatic interactions) necessitates more aggressive approaches. This includes developing cathode materials that can screen the strong Coulombic interactions of Mg^2+^ such as those with highly delocalized electronic states or materials exhibiting facile solid-stated diffusion of Mg^2+^ through unique crystallographic sites. Furthermore, designing heterogeneous interfaces with embedded nanoscale catalysts that provide energetically favorable pathways for multi-electron oxygen transfer and Mg^2+^ desolvation/insertion is essential [147]. This could involve using highly porous architectures to maximize active sites and minimize diffusion limitations, coupled with the introduction of catalytic dopants or surface modifiers that lower the activation energy for Mg^2+^ migration and oxygen redox. Across all three chemistries, incorporating strategies for managing local electric fields at the triple phase boundary (solid electrolyte, active material, and oxygen/gas phase) is also vital for facilitating efficient charge transfer and promoting faster ORR/OER.

While Na-ion, K-ion, and Mg-ion solid-state battery systems hold great promise for the future of energy storage, significant scientific and engineering challenges related to ionic conductivity, interfacial stability, material compatibility, mechanical integrity, and dendrite suppression need to be overcome for their widespread adoption.

### 4.2. Future Perspectives in Materials Innovation

Looking ahead, the future of non-lithium SSBs is inherently linked to the pace of material innovation, the development of novel fabrication processes, and the integration of these technologies into the broader energy ecosystem. Key areas of focus will include the discovery of new electrolyte and electrode materials that offer superior performance and safety profiles. Additionally, the role of computational modeling and machine learning in accelerating materials discovery and optimizing battery designs cannot be overstated. The integration of non-lithium SSBs into renewable energy systems, electric vehicles, and portable electronics presents a transformative opportunity to reshape our energy landscape. Success in this endeavor will require a concerted effort from academia, industry, and government to foster the development of these technologies, facilitate their commercialization, and implement policies that support the transition to a more sustainable and energy-efficient world.

#### 4.2.1. Polyanion-Type Cathode Materials for Na-Ion Solid-State Batteries

Intensifying research on the electronic conductivity and capacity of polyanion-type cathode materials is essential for the evolution of sodium-ion batteries. Strategies such as doping, which involves introducing elements capable of increasing charge carriers or altering electronic structures for enhanced electron mobility, are critical. Composite formation, which combines polyanion materials with conductive matrices, not only boosts overall conductivity, but also augments mechanical strength and structural integrity, essential for the durability of sodium-ion batteries [148]. Additionally, meticulous control over the morphology of these cathode materials can substantially enhance their performance. By optimizing the particle size and shape, researchers can greatly expand the interface area with the electrolyte, which in turn, facilitates faster and more efficient electrochemical reactions. Investigating novel polyanion structures and their intrinsic electrochemical properties is another frontier: such research might reveal cathode materials that exhibit exceptional capacity and stability, outperforming the current standards, and thereby increasing the cycle life. These advances could transform polyanion-type materials into leading candidates for high-performance sodium-ion batteries, offering improved energy storage solutions that are crucial for sustainable technology development.

#### 4.2.2. Phosphate-Based Cathode Materials for Na-Ion Solid-State Batteries

Enhancing the Na+ ion diffusion coefficient in NaFePO_4_ is vital for maximizing its utility as a superior cathode material in sodium-ion batteries. Efforts to improve this property include strategic doping with elements such as magnesium or aluminum, which can alter the electronic and ionic conductivity by modifying the crystal lattice structure. Nanostructuring is another effective approach that increases the electrode’s surface area, thus providing shorter paths for Na^+^ movement and potentially accelerating the charge–discharge rates. Interfacial engineering focuses on optimizing the contact points between the electrode and electrolyte, reducing resistance and improving ion transfer rates. Moreover, adopting advanced synthesis techniques tailored to promote the triphylite phase of NaFePO_4_ can lead to significant enhancements in electrochemical performance. These methods may involve controlled thermal treatments or the use of specific chemical precursors that encourage the formation of a more open crystal structure conducive to ion transport. By integrating these strategies, it is possible to develop NaFePO_4_ cathodes that not only exhibit improved rate capabilities and cycling stability, but also contribute to the development of sodium-ion batteries with higher energy densities and enhanced durability [149].

#### 4.2.3. Pyrophosphates as Cathode Materials for Na-Ion Solid-State Batteries

Key factors for enabling pyrophosphates as cathode materials for Na-ion solid-state batteries encompass a strategic focus on structure optimization, electrochemical performance enhancement, and computational studies. Structure optimization involves not only exploring structural modifications, but also implementing doping strategies aimed at improving electronic conductivity and Na^+^ diffusion, with a particular emphasis on Mn-containing pyrophosphates. This involves tweaking the crystal lattice and introducing dopants that can facilitate faster ion movement and increase electrical conductivity. Electrochemical performance enhancement focuses on extending the voltage stability, improving the cycle life, and enhancing the rate capability, which are critical for practical battery applications. These improvements are pursued through advanced synthesis techniques such as sol–gel processes or solid-state reactions, which allow for better control of the material’s microstructure, directly influencing its performance characteristics. Additionally, computational studies are increasingly vital, employing theoretical calculations to predict the most effective material compositions and structures. These studies help in guiding the synthesis and optimization efforts by providing insights into the material properties at an atomic level, thus enabling targeted improvements in the cathode materials [150].

#### 4.2.4. Transition Metal Sulfates of Na_x_M_y_ (SO_4_)_z_ (M = Fe, Mn, Co, Ni) for Na-Ion Solid-State Batteries

Key factors for enabling transition metal sulfates (Na_x_M_y_ (SO_4_)_z_) as cathode materials for Na-ion solid-state batteries include a multifaceted approach to material innovation and optimization. First, exploring diverse transition metal combinations, such as investigating the electrochemical properties of mixed-metal sulfates such as NaFeMn(SO_4_)_3_, is crucial. This strategy leverages the synergy between different transition metals to potentially achieve higher capacities and operating voltages. Second, optimizing Na^+^ ion transport through structural modifications, including doping or nanostructuring, is essential for creating more open frameworks that enhance Na^+^ ion diffusion pathways within the material. This leads to improved rate capability and cycling stability. Third, understanding and mitigating structural transformations during electrochemical cycling is vital. This involves unveiling the mechanisms behind structural changes and developing strategies to minimize unwanted phase transitions, thereby ensuring long-term stability and reliable performance. Additionally, in computational modeling and design, utilizing tools like DFT plays a critical role in predicting the optimal structures and guiding the design of novel transition metal sulfates with tailored properties. By addressing these key areas, transition metal sulfates hold immense promise for advancing the technology of sodium-ion batteries and enabling the development of high-energy, durable, and sustainable energy storage solutions [151].

#### 4.2.5. Transition Metal Silicates of Na_2_MSiO_4_ (M = Fe, Mn, Co) for Na-Ion Solid-State Batteries

Key factors for enabling transition metal silicates in Na-ion solid-state batteries include a multifaceted approach to improve their performance and viability. Synthesis optimization is crucial, with alternative methods such as hydrothermal synthesis and co-precipitation being explored to enhance the phase purity, morphology, and crystallinity of Na_2_MSiO_4_ materials. Doping and substitution strategies involve the introduction of transition metals or other elements into the Na_2_MSiO_4_ structure to enhance electronic conductivity, cyclability, and overall capacity. Additionally, electrolyte optimization focuses on selecting electrolytes that are electrochemically stable with Na_2_MSiO_4_ to minimize interfacial resistance. The design of composite electrodes that integrate Na_2_MSiO_4_ with conductive matrices such as carbon or graphene is also pivotal, as it enhances electron transport and facilitates Na^+^ ion diffusion, thereby improving the rate capability. Computational modeling, including the use of DFT and other tools, is employed to understand the intricate structure–property relationships in Na_2_MSiO_4_, guiding the design of optimized materials with targeted properties. By addressing these critical areas, orthosilicate transition metal silicates hold substantial promise for development into high-performance, sustainable, and cost-effective cathode materials for next-generation sodium-ion batteries [152].

#### 4.2.6. Further Classes of Cathode Materials for Na-Ion Solid-State Batteries

Expanding the range of cathode materials for sodium-ion solid-state batteries is essential for advancing the performance boundaries of these energy storage systems in the future. NASICONs, with their robust ionic conductivity and structural stability, are particularly promising for current and future sodium-ion battery applications. Organic compounds, on the other hand, offer flexibility in molecular design, which could lead to customizable electrochemical properties and novel functionalities. Hexacyanometalates also present interesting opportunities due to their open framework structures, which facilitate fast ion transport and can be fine-tuned through metal substitutions to optimize their electrochemical behavior. Moreover, the exploration of inorganic–organic hybrid materials represent a cutting-edge approach in the development of cathode materials [153]. These hybrids combine the high ionic conductivity and structural integrity of inorganic materials with the versatility and tailorability of organic compounds, potentially leading to cathode materials with synergistic properties. Such hybrids could deliver enhanced electrochemical performance, improved thermal stability, and reduced costs, making them highly suitable for future sodium-ion battery technologies. By integrating the strengths of both material classes, researchers can potentially unlock new pathways for energy storage solutions, paving the way for the development of more efficient, durable, and cost-effective sodium-ion batteries.

#### 4.2.7. Future Direction with Inorganic Solid Electrolytes for Na-Ion Solid-State Batteries

Key factors in the implementation of ISEs for sodium-ion solid-state batteries are multifaceted, focusing on enhancing mechanical flexibility, reducing interfacial resistance, and improving ionic conductivity. Composite electrolytes, which integrate ISEs with flexible polymer matrices, show great potential in achieving these goals. This approach not only improves mechanical flexibility, but also enhances the interfacial compatibility, maintaining high ionic conductivity crucial for efficient battery operation. Interface engineering, through surface modifications and designing interlayers, is also vital in minimizing interfacial resistance and ensuring long-term stability. Additionally, doping and substitution strategies within ISEs can modify bond energies, potentially increasing ionic conductivity and overall battery performance. Despite these advances, challenges such as brittleness, interfacial issues, and the limited ionic conductivity of ISEs still necessitate extensive research. By continuing to explore innovative composite designs, interface engineering, and advanced doping techniques, researchers can fully unlock the capabilities of ISEs, paving the way for compact, high-energy-density sodium-ion batteries suitable for a wide range of applications [154].

#### 4.2.8. Future Directions for K-Ion Solid-State Batteries

The advancement of potassium K-ion solid-state batteries is a dynamic research area focusing on overcoming key challenges to improve their performance, safety, and commercial applicability. Future research efforts are pivoting toward several promising directions. Researchers are exploring the creation of new solid electrolytes characterized by high ionic conductivity and exceptional chemical stability when in contact with potassium metal. These materials aim to facilitate efficient ion transport while preventing degradation reactions that compromise battery life. Given the large ionic radius of K^+^ ions, there is a concerted effort to design cathode materials that can not only accommodate these ions without significant structural changes during battery operation, but also maintain high energy density and stability through numerous charging cycles. Enhancing the electrode–electrolyte interface through innovative engineering techniques is crucial for improving ion transfer efficiency and minimizing resistance. This involves the strategic manipulation of material surfaces to promote seamless ion exchange and reduce potential bottlenecks in ion flow. The design of nanostructured electrode materials is under investigation to address issues like dendrite formation—a common cause of short circuits and battery failure. Nanostructuring aims to promote a uniform ion distribution, thereby improving battery safety and extending its operational life. Finally, alongside cathode development, the exploration of stable and high-performance anode materials that are compatible with potassium ions is critical. This includes materials that can undergo reversible K^+^ insertion/extraction processes without significant volume changes or degradation. The collective goal is to harness the inherent advantages of K solid-state batteries, such as material abundance, low cost, and potential for high energy density, making them a pivotal technology for future energy storage systems ranging from portable electronics to electric vehicles and grid storage solutions [130].

#### 4.2.9. Future Directions for Mg-Solid State Batteries

Advancement in Mg-ion solid-state batteries hinges on the development of solid electrolytes with high ionic conductivity, like sulfides, oxides, and halides, which are well-suited for Mg anodes. A key focus for researchers is synthesizing these solid electrolytes to enhance Mg^2+^ ion mobility and ensure stability against dendrite formation, which is crucial for maintaining the integrity and safety of batteries. The technique of interfacial engineering has also been explored to optimize the interaction between the electrolyte and electrodes, aiming to reduce the interfacial resistance and ensure uniform ion distribution, which are vital for consistent battery performance. On the cathode side, the emphasis is on identifying materials that can support the reversible intercalation of Mg^2+^ ions without significant structural changes or degradation, ensuring long-term durability and functionality. Materials such as transition metal oxides, sulfides, and organosulfur compounds are under scrutiny for their ability to retain capacity and support high discharge rates. Additionally, nanostructured materials are being explored to increase the surface area, which can significantly enhance the kinetic processes involved in ion transport, and consequently, the overall battery performance [155]. Composite electrolytes that merge polymers with inorganic fillers are also being developed to combine the high ionic conductivity of inorganic materials with the mechanical flexibility of polymers. This hybrid approach addresses the inherent brittleness of traditional solid electrolytes and enhances the mechanical stability of Mg solid-state batteries, offering a promising route to improve both safety and performance. By leveraging these diverse strategies, there is significant potential to push the boundaries of Mg-ion battery technology, paving the way for more efficient, stable, and robust energy storage solutions.

## 5. Summary and Conclusions

**Figure 7:**
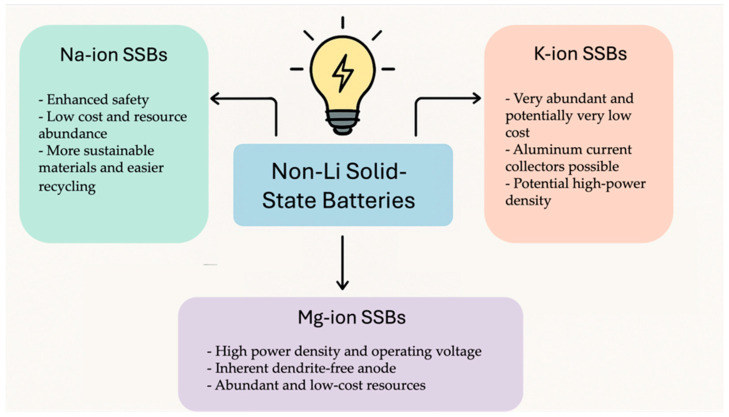
Advantages of Na-ion, K-ion, and Mg-ion solid-state batteries.

As we move further into the 21st century, finding reliable and sustainable ways to store energy is a top priority for scientists, industry leaders, and governments around the world. Better batteries with high energy and improved safety are key to switching to clean electricity and reaching a carbon-free future. Today’s lithium-ion batteries are widely used, but they still face problems like limited storage capacity, design challenges at the electrodes and interfaces, and rising material costs that can change quickly depending on the volatile market conditions. This review looked into the growing field of non-lithium solid-state batteries, which shows a lot of promise, as well as the considerable hurdles that lie ahead. We’ve highlighted some of the progress made so far, as well as the main challenges that still need to be resolved. The journey of non-lithium SSBs from laboratory level to market-ready, scaled-up manufacturing is far from straightforward. Issues such as enhancing the ionic conductivity, improving interfacial stability, and developing cost-effective and scalable manufacturing techniques remain significant barriers. Moreover, the sustainability of materials and the life cycle analysis of these batteries need to be thoroughly addressed to ensure their environmental viability. In conclusion, Na-ion, K-ion, and Mg-ion solid-state batteries present promising alternatives due to the wide availability and low cost of their constituent elements (Figure 7), which are more evenly distributed globally compared to lithium. Additionally, these systems offer intrinsic safety benefits and the potential for high volumetric capacities—particularly in the case of multivalent ions like magnesium—making them strong candidates for grid-scale storage and next-generation portable devices.

In conclusion, while the road ahead is challenging, the potential rewards of non-lithium solid-state batteries are immense. By continuing to develop better materials and smarter designs, we can look forward to a future where these new batteries can play a pivotal role in driving forward the green energy revolution and building a more sustainable and electrified future.

## Figures and Tables

**Table 1:** The comparison between LTMOs, polyanionic compounds, and PBA materials. Notes: (1) Volumetric energy density (Wh/L) can be approximated by multiplying the gravimetric energy density (Wh/kg) by the tap density (kg/L) of the active material. For example, NaFePO_4_ with a gravimetric energy density of ~110 Wh/kg and a tap density of ~2.0 kg/L yields an estimated volumetric energy density of ~220 Wh/L, which is an idealized case assuming dense electrode packing. In practical battery electrodes, porosity, binder and additive content, and inactive components significantly reduce the effective volumetric energy density. (2) A number of studies have reported electrode-based energy densities at higher values (particularly gravimetric), often measured at the material or electrode level under idealized lab conditions. The third column below conservatively reflects practical energy density ranges, accounting for full-cell configurations, real-world packing densities, and electrode architecture constraints. (3) Due to the intrinsically low tap density of PBAs with open framework architectures, current commercial Na-ion batteries typically demonstrate volumetric energy densities of ~50 Wh/L [26]. With optimized electrode engineering and compaction strategies, higher densities might be possible. (4) CATL and Northvolt reported Na-ion gravimetric energy densities of 160–175 Wh/kg in full-cell prototypes [27,28].

Type	Material	Advantage	Disadvantage	Specific Energy Density
LTMO	O2- and P3-type layered structures composed of transition metals (e.g., Mn, Fe, Co)	- High specific capacity enabling energy-dense applications - High electrochemical potential versus Na^+^/Na - Straightforward and cost-effective synthesis	- Poor structural integrity under repeated cycling - Limited long-term storage stability - Sluggish sodium-ion diffusion kinetics	~100–170 Wh/kg (gravimetric) ~250–375 Wh/L (volumetric) [29,30,31,32]
Polyanionic compounds	Polythiophene-modified NaFePO_4_ and maricite-phase NaFePO_4_ structures	- High thermal and chemical stability, suitable for high-temperature applications - Elevated redox potential due to strong inductive effects of polyanions	- Intrinsically low electronic conductivity, requiring conductive coatings or additives - Relatively low gravimetric energy density	~100–150 Wh/kg (gravimetric) ~200–300 Wh/L (volumetric) [33,34,35]
PBAs	Open-framework cubic structures, typically Na_1.92_Fe_2_(CN)_6_ with Fe(CN)_6_ vacancies	- Low production cost due to readily available precursors - Simple and scalable synthesis methods - Competitive electrochemical performance in sodium-ion systems	- Susceptibility to structural instability during cycling - Presence of Fe(CN)_6_ vacancies that reduce electrochemical activity and capacity	~160–175 Wh/kg (gravimetric) ~50 Wh/L (volumetric) [26,27,28,32,36,37]

**Table 2:** The comparison of polymorphism structures with cell parameters and space groups of Na_2_MP_2_O_7_ (M = Fe, Co, Mn, Cu).

	Structure	a/Å	b/Å	c/Å	β/Å	Vol/Å	Ref.
Na_2_FeP_2_O_7_	Triclinic	6.4299	9.4145	11.0110	85.465	573.39	[58]
Na_2_CoP_2_O_7_	Triclinic	9.735	10.940	12.289	121.76	566.8	[62]
	Orthorhombic	15.4061	10.2885	7.7031	-	1221.0	[63]
	Tetragonal	7.706	10.301	-	-	-	[63]
Na_2_MnP_2_O_7_	Triclinic	5.316	6.580	9.409	95.25	290.96	[58]
β-Na_2_MnP_2_O_7_	Triclinic	9.922	11.086	12.473	121.94	599.4	[58]
α-Na_2_CuP_2_O_7_	Monoclinic	8.823	13.494	5.108	92.77	607.5	[63]
β-Na_2_CuP_2_O_7_	Monoclinic	14.728	5.698	8.067	115.15	612.8	[63]

**Table 4:** Comparison of solid polymer electrolytes.

Solid Polymer Electrolyte	Electrochemical Stability Window/V (vs. Na^+^/NA)	Ionic Conductivity	Ionic Transference Number	References
PEO-NaFNFSI	4.87	3.36 × 10^−4^ at 80 °C	0.24	[77]
PTMC-NaFSI	4.8	5 × 10^−5^ at 25 °C	0.48	[76]
PVCA-NaTf	5.3	1.2 × 10^−4^ at 25 °C	0.60	[78]
PCL-NaFSI	-	1.28 × 10^−4^ at 25 °C	0.50	[79]
NaPTAB-SGPE	4.8	1.43 × 10^−4^ at 60 °C	0.91	[91]

**Table 5:** The comparison between Na-ion, K-ion and Mg-ion SSBs.

Type	Materials	Advantage	Disadvantage
Na-Ion SSB	- Cathode: LTMO, polyanionic compounds, PBA - Anode: Na metal, carbon-based anodes - Electrolytes: Inorganic, composite, solid polymer	- Enhanced safety - Low cost and resource abundance - More sustainable materials and easier recycling	- Low energy density - Challenge in achieving high Na^+^ conductivity - Short cycle life
K-Ion SSB	- Cathode: PTCDA, - Anode: K metal, K-RGO - Electrolytes: Organic, inorganics, and hybrids	- Very abundant and potentially very low cost - Aluminum current collectors possible - Potential high-power density	- Low energy density - Sluggish ion kinetics in solids - More reactive potassium metal
Mg-Ion SSB	- Cathode: Carbon black - Anode: Mg metal - Electrolytes: Organic, inorganics, and hybrids	- High power density and operating voltage - Inherent dendrite-free anode - Abundant and low-cost resources	- Very challenging Mg^2+^ ion mobility in solids - High interfacial resistance - Limited electrolyte and electrode compatibility

## Abbreviations

ALD	Atomic layer deposition
ASSLB	All-solid-state lithium-ion battery
CBM	Conduction band minimum
CCD	Critical current density
CEI	Cathode electrolyte interphase
CNT	Carbon nanotube
CV	Cyclic voltammetry
DFT	Density functional theory
DMF	N,N-dimethylformamide
EBSD	Electron backscatter diffraction
EIS	Electrochemical impedance spectroscopy
EELS	Electron energy loss spectroscopy
EV	Electric vehicle
GB	Grain boundary
HAADF-STEM	High-angle annular dark-field scanning transmission electron microscope
HOMO	Highest occupied molecular orbital
ISE	Inorganic solid electrolyte
LATP	Li_1.3_Al_0.3_Ti_1.7_(PO_4_)_3_
LAGP	Li_1.5_Al_0.5_Ge_1.5_(PO_4_)_3_
LGPS	Li_10_GeP_2_S_12_
LIBs	Lithium-ion batteries
LiPON	Lithium phosphorus oxynitride
LISICON	Lithium superionic conductor
LPS	Li_3_PS_4_: Li_7_P_3_S_11_
LPSCl	Li_6_PS_5_Cl
LLZO	Li_7_La_3_Zr_2_O_12_
LTMO	Layered transition metal oxide
LTO	Li_4_Ti_5_O_12_
LUMO	Lowest unoccupied molecular orbital
LYBC	Li_3_Y(Br_3_Cl_3_)
LZO	Li_6_Zr_2_O_7_
NASICON	Sodium superionic conductor
NaClO_4_	Sodium perchlorate
NaFNFSI	(Sodium (fluorosulfonyl)(n-nonafluorobutanesulfonyl)imide)
NaPTAB	Sodium–Poly(tartaric acid) Borate
NMR	Nuclear magnetic resonance
PB	Prussian blue
PAN	Polyacrylonitrile
PBA	Prussian blue analog
PE	Polyethylene
PEO	Polyethylene glycol
PP	Polypropylene
P(VDF-HFP)	Poly(vinylidene fluoride-co-hexafluoropropylene)
RCC	Current constriction resistance
RCT	Charge transfer resistance
rGO	Reduced graphene oxide
Rint	Interfacial resistance
SEM	Scanning electron microscope
SEI	Solid electrolyte interphase
SGLE	Single-Ion Gel Polymer Electrolyte
SHE	Standard hydrogen electrode
SPE	Solid polymer electrolyte
SSB	Solid-state battery
SSE	Solid-state electrolyte
SSKB	Solid-state K-ion battery
SSMB	Solid-state Mg-ion battery
SSSB	Sodium-ion solid-state battery
TiO_2_	Titanium dioxide
VBM	Valence band maximum
XAS	X-ray absorption spectroscopy
XPS	X-ray photoelectron spectroscopy
